# PHF5A is a potential diagnostic, prognostic, and immunological biomarker in pan-cancer

**DOI:** 10.1038/s41598-023-44899-6

**Published:** 2023-10-16

**Authors:** Na Ding, Meiping Li, Xiaokun Zhao

**Affiliations:** 1 Department of Pathology, Shaoxing Maternity and Child Health Care Hospital, Shaoxing, 312000 Zhejiang People’s Republic of China; 2 School of Medicine, Shaoxing University, Shaoxing, 312000 Zhejiang People’s Republic of China

**Keywords:** Computational biology and bioinformatics, Biomarkers, Medical research

## Abstract

Studying the molecular mechanisms and regulatory functions of genes is crucial for exploring new approaches and tactics in cancer therapy. Studies have shown that the aberrant expression of PHF5A in tumors is linked to the origin and advancement of multiple cancers. However, its role in diagnosis, prognosis, and immunological prediction has not been comprehensively investigated in a pan-cancer analysis. Using several bioinformatic tools, we conducted a systematic examination of the potential carcinogenesis of PHF5A in various tumors from multiple aspects. Our analysis indicated that PHF5A expression varied between normal and tumor tissues and was linked to clinical diagnosis and prognosis in various cancers. The results confirmed a notable variation in the levels of PHF5A promoter methylation among several types of primary tumor and normal tissues and methylation of the PHF5A promoter played a guiding role in prognosis in some cancers. According to our findings, PHF5A played a critical role in tumor immunity and it might be an excellent target for anticancer immunotherapy. To sum up, PHF5A can be used in pan-cancer diagnostics, prognostics, and immunotherapy.

## Introduction

Cancer is consistently listed among the primary factors that contribute to mortality and a significant obstacle to improving human lifespan. The worldwide incidence of cancer is expected to rise significantly by 2040, with an estimated 28.4 million cases, indicating a substantial 47% increase compared to the recorded figures in 2020^1^. Currently, the primary cancer treatment modalities encompass surgical intervention, chemotherapy, targeted therapy, radiation therapy, and immunotherapy; however, the prognosis and survival outcomes for patients remain suboptimal^2^. Consequently, it is imperative to identify and characterize novel pan-cancer genes to enhance comprehension of the exceedingly intricate tumorigenesis process. Notably, several prospective novel hallmarks of cancer have been proposed^3^. Thereinto, the significance of epigenetic changes in cancer onset and progression has been paid increasing attention^4^, and aberrant splicing is widely recognized as a significant epigenetic characteristic of cancer^5^. Here we focus on a protein domain called PHF5A, which is closely involved in RNA splicing, and may be served as a valuable therapeutic target.

PHF5A, a protein that is highly conserved in the nucleus and widely expressed in eukaryotes, encodes a protein that has a characteristic zinc-finger domain called PHD. According to reports, PHF5A has a complex function as a general transcription activator for various genes^6^. Systematic deletion of PHF5A in yeast leads to lethality, and similarly, depletion of PHF5A in *C. elegans* during early morphogenetic phase results in aberrant organogenesis and embryonic lethality^7,8^. Moreover, PHF5A finally regulates the stem cell cellular reprogramming and pluripotency maintenance by directing the transcriptional program^9^. Furthermore, it can localize to promoters by recognizing histones and regulate splicing alterations across the genome^5^. Recent study shows that PHF5A promotes p400-mediated IgH locus chromatin remodeling, which impacts cis- and trans-recombination. Consequently, it plays a crucial role in regulating DNA repair during class switch recombination, thus earning it the classification of a chromatin regulator^10^. PHF5A is essential for maintaining the structural stability of the spliceosome and it has a vital function in linking the spliceosome to histones^8^. As response to cellular stresses, PHF5A can undergo acetylation at lysine 29. This acetylation enhances the interaction among U2 snRNPs, thereby affecting extensive gene expression as well as pre-mRNA splicing patterns^11^. The abnormal expression of PHF5A in tumors has been observed to be linked to the genesis and progression of cancer in numerous studies^5,11–15^. However, most studies on PHF5A are confined to one specific type of cancer. Consequently, conducting a comprehensive analysis of molecular mechanisms and regulatory functions of PHF5A across various types of cancer is crucial in order to offer new insights and approaches for cancer treatment.

In present study, we applied various bioinformatics methods to systematically analyze PHF5A in pan-cancer. Our analysis encompassed gene expression status, diagnosis value, survival prognosis, DNA methylation, molecular function, pathway enrichment, drug sensitivity and tumor immunity through combining data from different databases. Through our analysis, we gained a thorough comprehension of the roles that PHF5A played in the development and prognostication of various kinds of cancer. The findings have revealed that PHF5A may function as a diagnostic and prognostic factor for various tumor types. Moreover, it may impact significantly on tumor immunity and immunotherapy.

## Methods

### Analysis of PHF5A expression

PHF5A expression was conducted in both tumor and normal tissues utilizing TIMER2, which can be accessed at http://timer.cistrome.org/^16^. In order to evaluate the variations in PHF5A expression levels for cancers that have limited or unavailable normal tissue data, we employed the GEPIA2 online platform (http://gepia2.cancer-pku.cn/#index) by matching the GTEx database with TCGA^17^. Furthermore, The XIANTAO platform (https://www.xiantaozi.com/) was utilized to evaluate PHF5A expression in paired tumor as well as paracancerous tissues^18^.

We utilized GEPIA2 tool to acquire PHF5A expression at different clinicopathological stages in TCGA tumors. Using the TCGA dataset, we also conducted a comprehensive analysis of the expression levels of PHF5A in relation to tumor grade. Additionally, we analyzed the M and N stages of various tumors to investigate the underlying relationship between PHF5A and tumor metastasis. Analysis of variance (ANOVA) was used to conduct difference testing for multiple groups of samples.

The UALCAN tool was utilized to analyze protein expression by leveraging data from the CPTAC and ICPC databases and the protein expression for 11 tumor types is available^19,20^. The expression level of PHF5A protein has been compared between normal tissues and primary tumors, and Z-value indicates deviation from the median level in a specific cancer type. Additionally, differences in protein-level expression of PHF5A was also investigated using the HPA database^21^. We acquired and examined immunohistochemical (IHC) images of PHF5A protein manifestation in 12 tumor types. UALCAN tool is accessible through the website https://ualcan.path.uab.edu/index.html and HPA database is accessible through the website https://www.proteinatlas.org/.

What’s more, we employed TISIDB online tool to examine the correlations of PHF5A expression with molecular subtypes of 17 cancers, as well as immune subtypes of 30 cancers^22^. TISIDB database can be accessed at http://cis.hku.hk/TISIDB/index.php. The significance of discrepancy between two groups of samples was determined by Dunn’s test, while the statistical significance of multiple groups of samples was analyzed by the Kruskal–Wallis test.

### Analysis of diagnosis accuracy

The diagnostic accuracy of gene signature is often evaluated by AUC value, which represents the area under ROC curve (Receiver Operating Characteristic)^23^. Using R software with “pROC” package, we analyzed the ROC curve based on specificity and sensitivity of PHF5A^24^.

### Analysis of survival prognosis

By utilizing the ‘Survival Map’ functionality within GEPIA2 tool, we acquired the OS (overall survival) and DFS (disease-free survival) prognoses associated with various levels of PHF5A expression in distinct tumors. Patients with low and high expression were equally divided. Next, the ‘Survival Plots’ function was employed to examine Kaplan–Meier curves for particular cancers, where the presence of PHF5A demonstrated a significant association with survival prognosis. Moreover, to determine the significance of PHF5A in predicting OS, DFS, PFS (progression free survival) and DSS (disease-specific survival), the univariate cox regression analysis was conducted utilizing ‘survival’ and ‘forestplot’ R packages^25^. RNA sequencing and clinical data used were downloaded from TCGA dataset through the website https://portal.gdc.com.

### Analysis of DNA methylation

UALCAN online tool was also employed to research the methylation level of the PHF5A promoter in normal tissues and 24 different types of primary tumors. Beta value which ranges from 0 to 1 was used to characterize the DNA methylation level. Furthermore, GSCA platform was employed to study the correlations of the methylation level of PHF5A with prognosis, including OS, PFS, DSS and DFS^26^. GSCA database can be accessed at http://bioinfo.life.hust.edu.cn/GSCA/#/.

### Enrichment analysis

Initially, STRING tool^27^ was utilized to generate available experimentally determined Homo sapiens PHF5A-binding proteins under conditions with maximum of 50 interactors and low confidence. STRING tool is accessible through the website https://string-db.org/. Next, from all TCGA cancer types, the top 100 PHF5A expression-related genes were acquired using GEPIA2 tool. Then, the correlation of these selected genes with PHF5A was performed. Moreover, the correlation heatmap of PHF5A with the 5 selected genes in each tumor was obtained using TIMER2. Afterwards, these two datasets were then merged for GO and KEGG enrichment analysis using “clusterProfiler” R packages^28^. In addition, the LinkInterpreter functionality of the LinkedOmics platform was utilized to independently conduct the GSEA analysis of KEGG pathway in 32 types of tumors using genes associated with PHF5A^29^. LinkedOmics platform can be accessed at http://linkedomics.org/admin.php.

### Immunological analysis

We evaluated the correlation of immune scores with PHF5A expression using the “immunedeconv” R package, which includes six algorithms such as xCell and TIMER, using RNA sequencing expression profiles^30^. To assess the statistical distinction, the Wilcoxon test was performed. Furthermore, we used the SangerBox platform (http://sangerbox.com, version 3.0) to conducted the co-expression analysis of PHF5A with immune checkpoint genes, chemokine receptor genes, chemokine genes, as well as major histocompatibility complex (MHC)^31^. The results were visualized by ‘RColorBrewer’ and ‘reshape2’ R packages. The relationship of PHF5A expression with tumor mutation burden (TMB), microsatellite instability (MSI), neoantigens (NEO) as well as homologous recombination deficiency (HRD) in various tumors were also conducted by the SangerBox online platform and visualized by radar maps using “ggplot2” R software package.

### Drug sensitivity analysis

We carried out an investigation to explore a possible correlation between sensitivity to anti-tumor drugs and PHF5A expression. To accomplish this, we employed the GSCA platform, which integrates data from the GDSC source. The drug sensitivity was assessed using the IC50 methodology.

## Results

### PHF5A expression analysis

**Figure 1:**
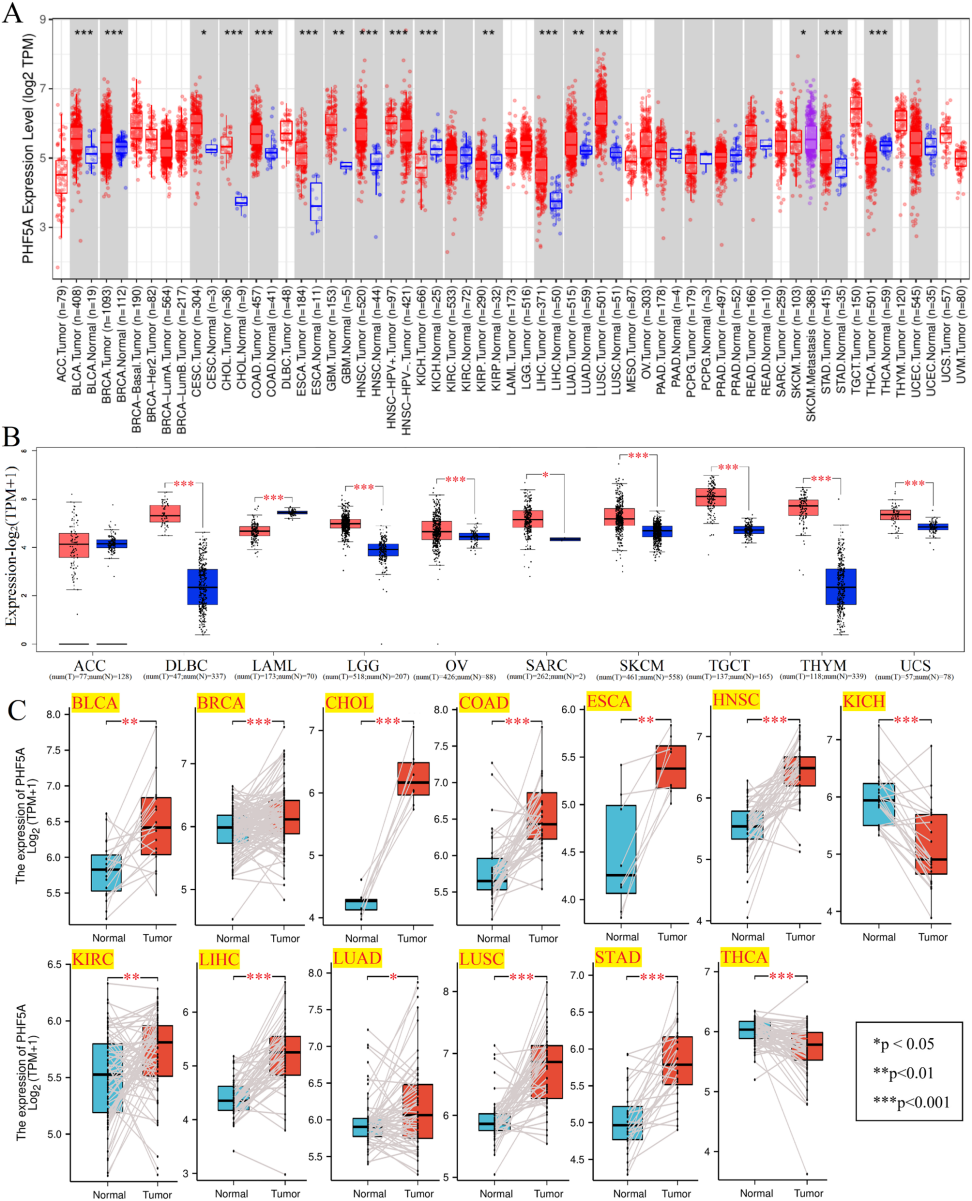
PHF5A expression in various kinds of cancer. (**A**) PHF5A expression in various kinds of tumor using TIMER2 tool. (**B**) PHF5A expression for ACC, DLBC, LAML, LGG, OV, SARC, SKCM, TGCT, THYM and UCS by matching GTEx database with TCGA. (**C**) PHF5A expression in paired tumor and paracancerous tissues. *p < 0.05; **p < 0.01; ***p < 0.001.

Figure 1A demonstrated that PHF5A expression level was compared between tumor and corresponding normal tissues using TIMER2 tool. As compared to normal tissues, PHF5A expression was considerably elevated in Bladder urothelial carcinoma (BLCA), Breast invasive carcinoma (BRCA), Cholangiocarcinoma (CHOL), Colon adenocarcinoma (COAD), Esophageal carcinoma (ESCA), Head and neck squamous cell carcinoma (HNSC), Liver hepatocellular carcinoma (LIHC), Lung squamous cell carcinoma (LUSC), Stomach adenocarcinoma (STAD), Glioblastoma multiforme (GBM), Lung adenocarcinoma (LUAD) and Cervical squamous cell carcinoma and endocervical adenocarcinoma (CESC) with a p-value of less than 0.05. In the meantime, there was a notable reduction in PHF5A expression in Kidney renal papillary cell carcinoma (KIRP), Kidney chromophobe (KICH) and Thyroid carcinoma (THCA) (p < 0.05). Then, the GTEx database was matched to TCGA database to assess the differences in PHF5A expression in tumors with limited or no available normal tissue data. Figure 1B showed the PHF5A expression in tissues of Lymphoid neoplasm diffuse large B-cell lymphoma (DLBC), Brain lower grade glioma (LGG), Ovarian serous cystadenocarcinoma (OV), Skin cutaneous melanoma (SKCM), Testicular germ cell tumors (TGCT), Thymoma (THYM), Uterine carcinosarcoma (UCS) and Sarcoma (SARC) was also significantly increased. Conversely, a notable decreasing expression of PHF5A was also observed in Acute myeloid leukemia (LAML) tissues. Since both Mesothelioma (MESO) and Uveal melanoma (UVM) also lack corresponding normal tissue data in the GTEx database, these two cancer types were not included or compared in Fig. 1B. For paired tumor and paracancerous tissues, aside from some tumors lacking paired data, PHF5A demonstrated high expression levels in BLCA, BRCA, CHOL, COAD, ESCA, HNSC, Kidney renal clear cell carcinoma (KIRC), LIHC, LUAD, LUSC and STAD (Fig. 1C). Conversely, it exhibited low expression in KICH and THCA. The findings were consistent with above results (Fig. 1A,B).

**Figure 2:**
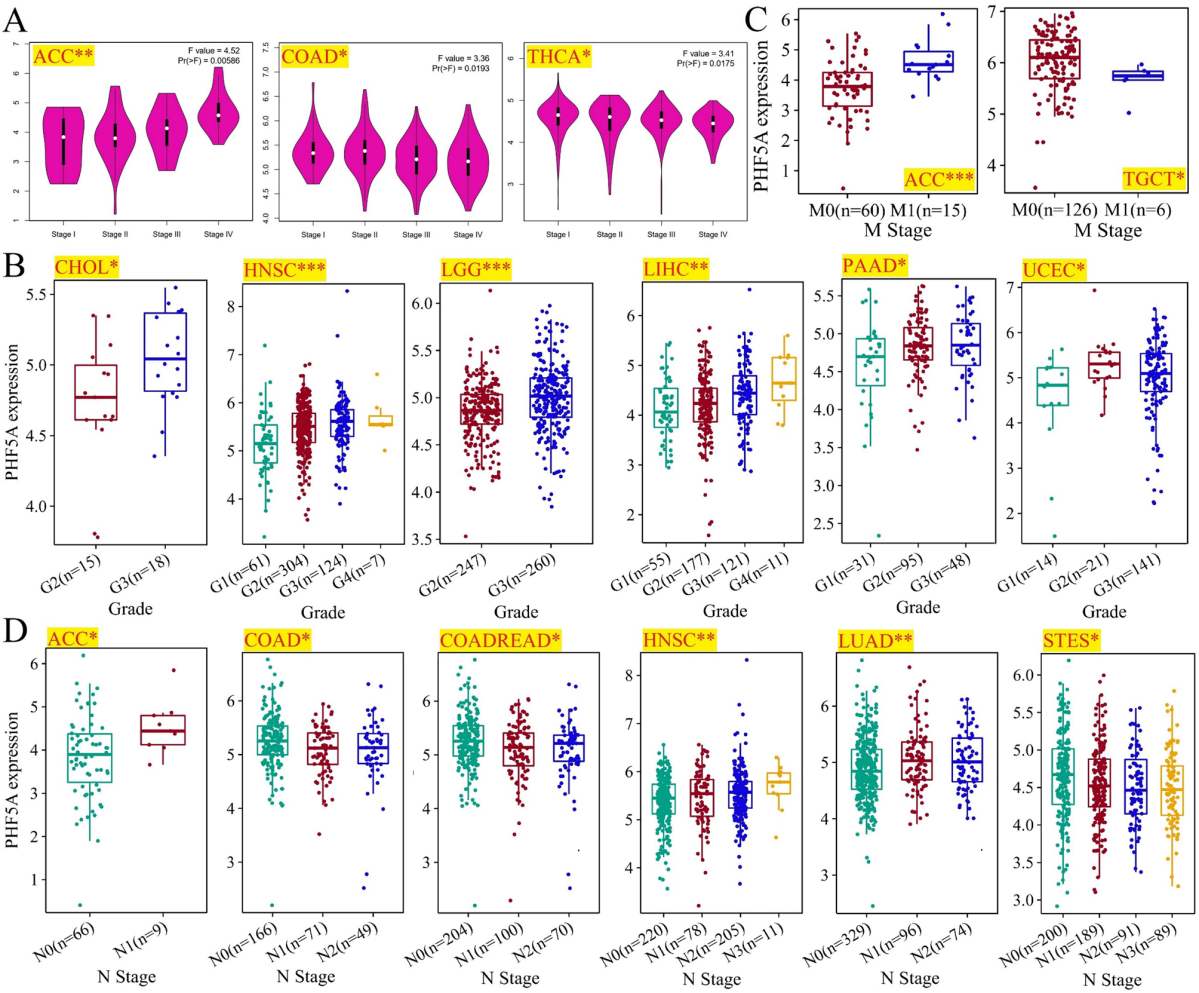
Correlation between clinical staging of tumors and PHF5A expression. All significant results from TCGA tumors were presented. (**A**) The relationship between main pathological stages of ACC, COAD and THCA and PHF5A expression. Log2 (TPM + 1) was used to convert the data to a logarithmic scale. (**B**) Correlation of tumor grade (CHOL, HNSC, LGG, LIHC, PAAD and UCEC) with PHF5A expression. (**C**) Correlation of M-stage with PHF5A expression in ACC and TGCT. (**D**) Correlation of tumor N-stage (ACC, COAD, COADREAD, HNSC, LUAD and STES) with PHF5A expression. *p < 0.05; **p < 0.01; ***p < 0.001.

Moreover, we also utilized GEPIA2 tool to acquire PHF5A expression at different clinicopathological stages in TCGA tumors. According to Fig. 2A, the correlation of clinicopathological stages with PHF5A expression was observed in Adrenocortical carcinoma (ACC), COAD, and THCA with a significance level of p-value less than 0.05. While, no notable association was observed in other types of cancer that we studied. In ACC, we can see a strong correlation of elevated PHF5A expression with the advanced stage. However, in COAD and THCA, higher stages were associated with reduced expression levels of PHF5A. Furthermore, we investigated the connection between PHF5A expression and tumor grade. Due to the absence of tumor grading information for certain cancers, we only obtained data for 13 cancer types from TCGA. Among these, we noticed a substantial connection between tumor grade and PHF5A expression in 6 cancer types, including CHOL, HNSC, LGG, LIHC, PAAD and UCEC (Fig. 2B). Upon analysis of these 6 tumor types, it was evident that the expression of PHF5A corresponded directly with the tumor grade. As the PHF5A expression increased, the tumor grade also developed in an upward direction. In addition, the relevance between PHF5A expression and tumor metastasis was preliminarily investigated. Firstly, we analyzed tumors with M-stage data in TCGA and discovered a notable correlation of M-stage with PHF5A expression in 2 cancer types, namely ACC and TGCT (Fig. 2C). In ACC, PHF5A expression increased when the tumor had distant metastasis, whereas in TGCT, PHF5A expression decreased when the tumor had distant metastasis. Afterwards, we examined the expression of PHF5A according to N staging and discovered a significant association of lymph node metastasis with PHF5A expression in 6 types of cancer: ACC, COAD, Colon adenocarcinoma/Rectum adenocarcinoma esophageal carcinoma (COADREAD), HNSC, LUAD, and Stomach and esophageal carcinoma (STES) (Fig. 2D).

**Figure 3:**
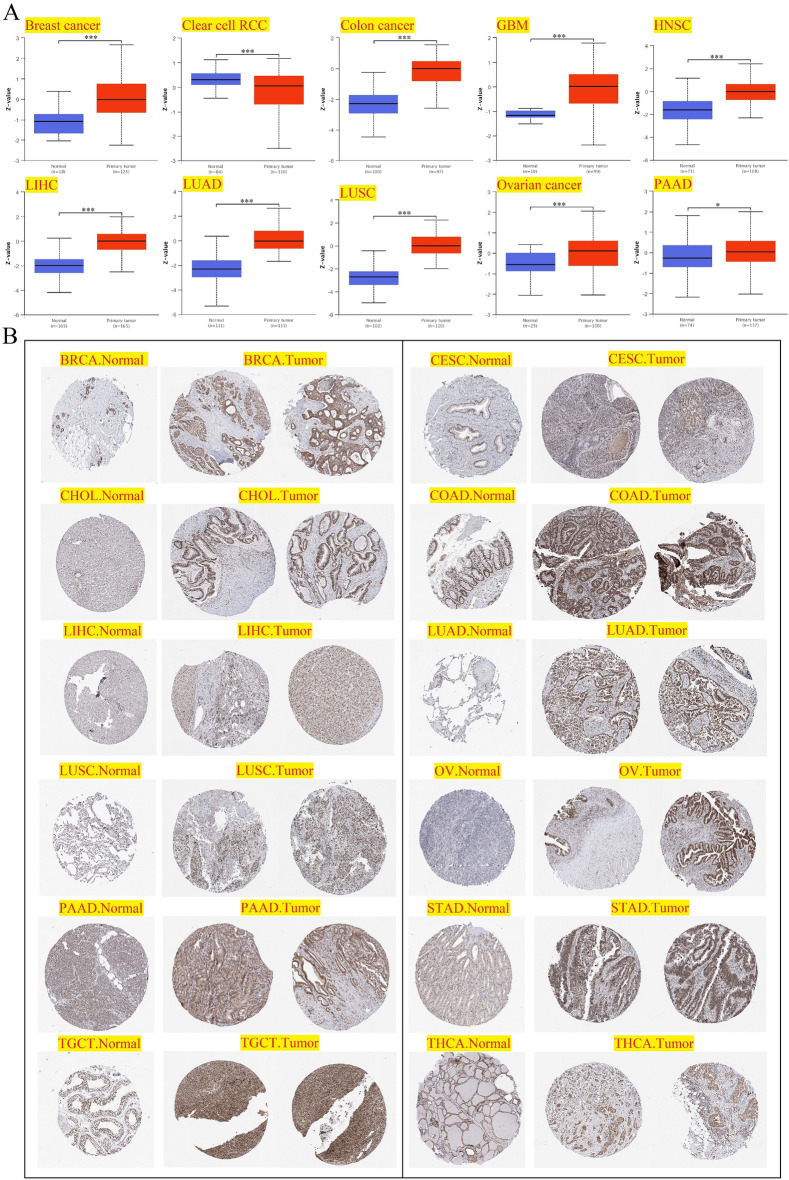
The PHF5A protein expression level. All significant results were presented. (**A**) PHF5A total protein in both tumor and normal tissues for various cancers using UALCAN tool. *p < 0.05; **p < 0.01; ***p < 0.001. (**B**) IHC images of PHF5A expression in both normal and tumor groups in various kinds of cancer.

The analysis results of PHF5A total protein expression were shown in Fig. 3A. Compared to normal tissues, primary tumor tissues of breast cancer, colon cancer, GBM, HNSC, LIHC, LUAD, LUSC, ovarian cancer and Pancreatic adenocarcinoma (PAAD) showed considerably higher expression of PHF5A total protein. By contrast, clear cell renal cell carcinoma (clear cell RCC) was found a lower PHF5A total protein when compared to normal tissues. Then, we utilized the HPA online tool to extract the IHC images for further evaluation of PHF5A protein expression. The higher protein expression of PHF5A, compared to normal tissues, can be observed intuitively in BRCA, CESC, CHOL, COAD, LIHC, LUAD, LUSC, OV, PAAD, STAD, and TGCT (Fig. 3B). And THCA was found a lower protein expression of PHF5A when compared to normal tissues. The IHC images provided results that were compatible with PHF5A expression data obtained in Fig. 1.

**Figure 4:**
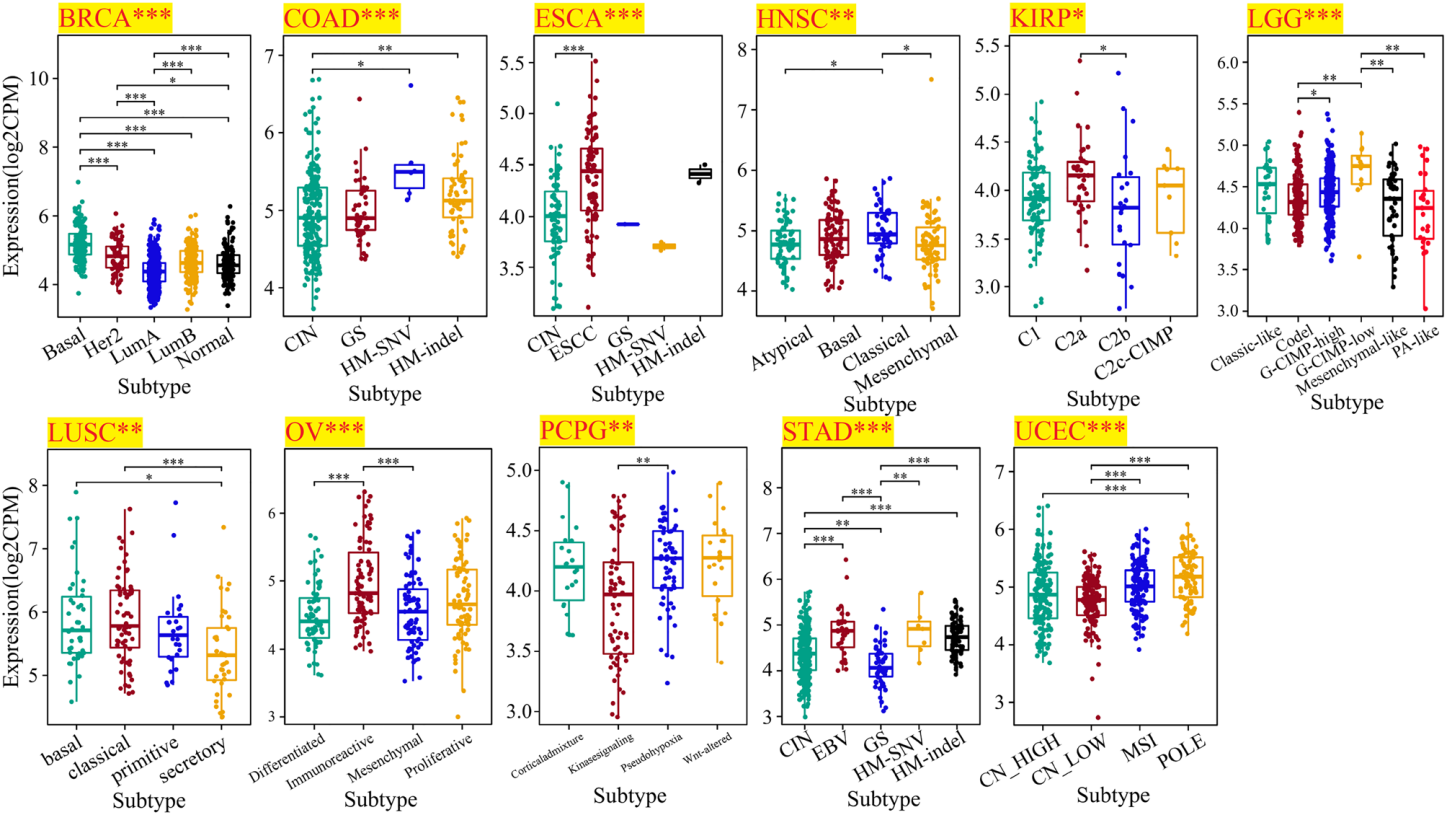
The correlation of PHF5A expression with molecular subtypes in various cancers. All significant results from TCGA tumors were presented. The significance of discrepancy between two groups of samples was determined by Dunn’s test, while the statistical significance of multiple groups of samples was analyzed by the Kruskal–Wallis test. *p < 0.05; **p < 0.01; ***p < 0.001.

**Figure 5:**
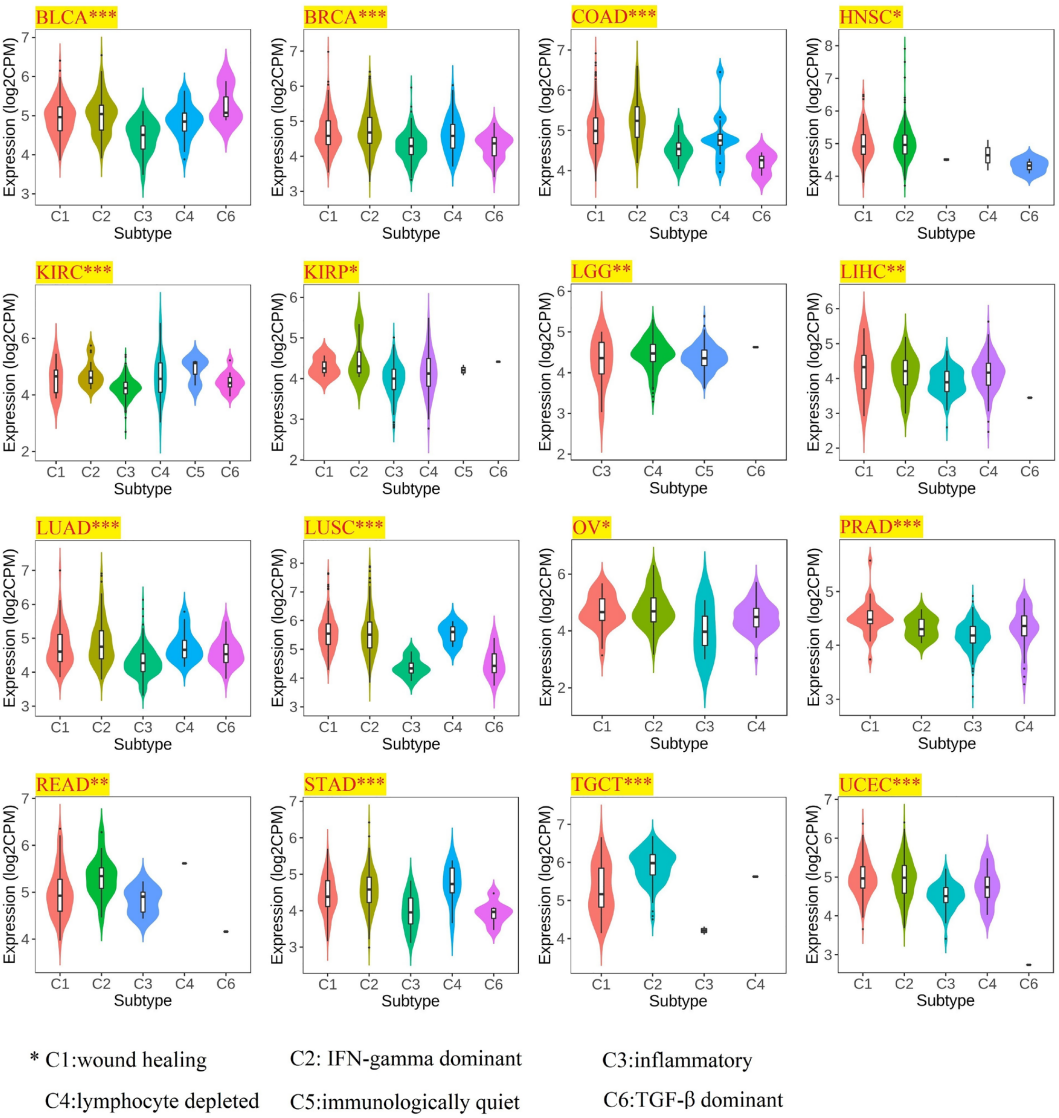
The correlation of PHF5A expression with immune subtypes in various cancers. All significant results from TCGA tumors were presented. The statistical significance of any discrepancies was analyzed by Kruskal–Wallis test. *p < 0.05; **p < 0.01; ***p < 0.001.

Subsequently, correlation of molecular and immune subtypes in cancers with PHF5A expression was demonstrated. Figure 4 revealed a considerably correlation of PHF5A expression with various molecular subtypes in BRCA, COAD, ESCA, HNSC, KIRP, LUSC, LGG, OV, Pheochromocytoma and paraganglioma (PCPG), STAD and Uterine corpus endometrial carcinoma (UCEC). Figure 5 revealed a notably correlation of PHF5A expression with various immune subtypes in BLCA, BRCA, COAD, HNSC, KIRC, KIRP, LGG, LUSC, LUAD, LIHC, OV, Prostate adenocarcinoma (PRAD), Rectum adenocarcinoma (READ), STAD, TGCT and UCEC. Thus, we can conclude that PHF5A expression varies across molecular and immune subtypes in different kinds of cancer.

### Diagnosis accuracy of PHF5A across various tumors

**Figure 6:**
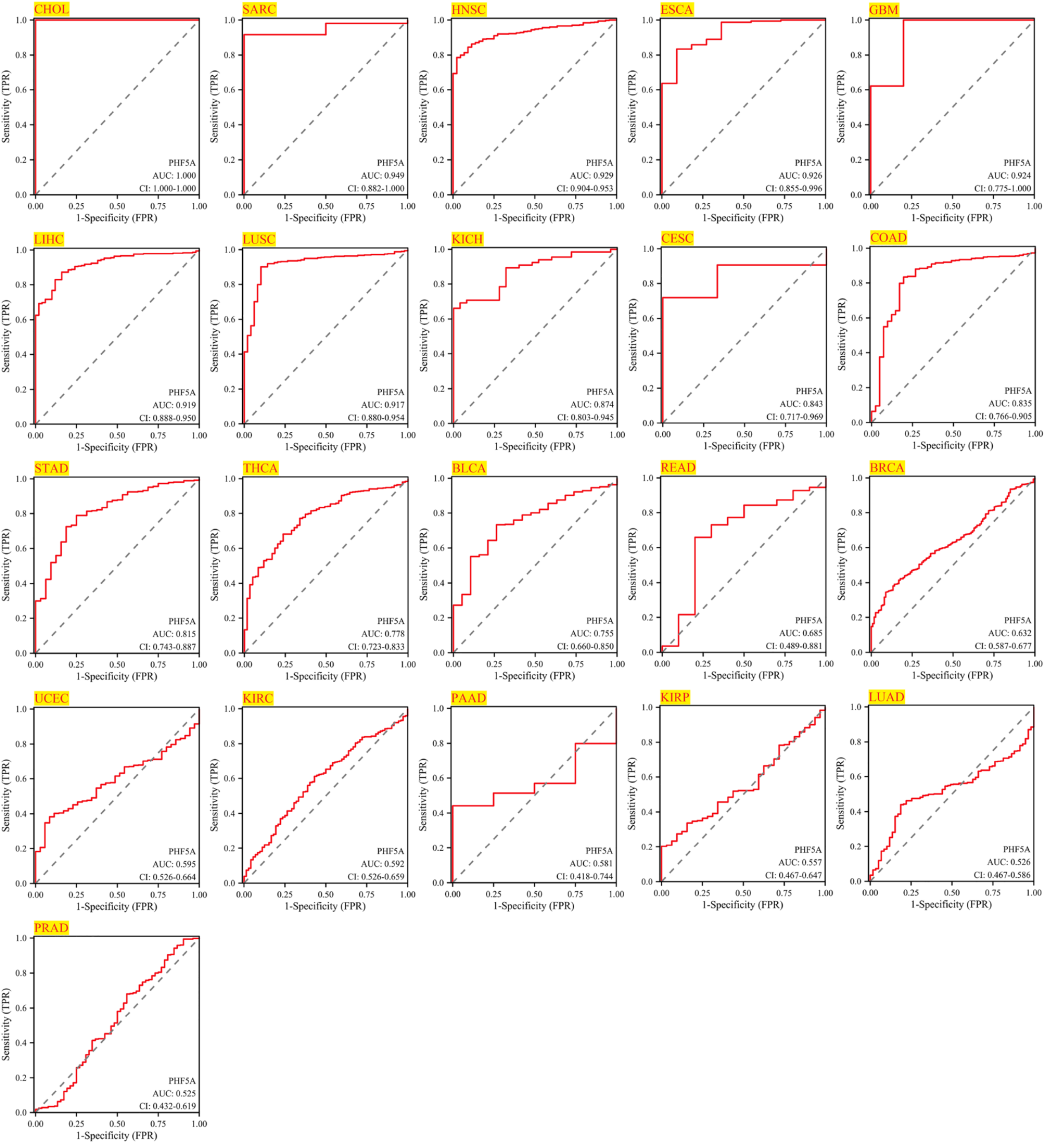
Diagnostic value of PHF5A for CHOL, SARC, HNSC, ESCA, GBM, LIHC, LUSC, KICH, CESC, COAD, STAD, THCA, BLCA, READ, BRCA, UCEC, KIRC, PAAD, KIRP, LUAD and PRAD using ROC curve.

The AUC value typically falls within the range of 0.5 and 1.0. The accuracy of the variable in predicting outcomes improves when the AUC value approaches 1.0, thereby enhancing its diagnostic effectiveness. The AUC cutoff value of 0.5 to 0.7 indicates lower accuracy, 0.7 to 0.9 suggests relative diagnostic accuracy, and values of 0.9 to 1.0 indicate higher diagnostic accuracy^23^. The results demonstrated that AUC value of PHF5A had low diagnostic accuracy for 8 cancer types (including READ, BRCA, UCEC, KIRC, PAAD, KIRP, LUAD and PRAD), relative diagnostic accuracy for 6 cancer types (including KICH, CESC, COAD, STAD, THCA, BLCA), and high diagnostic accuracy for 7 cancer types (including CHOL, SARC, HNSC, ESCA, GBM, LIHC and LUSC) (Fig. 6). An important point to note was that AUC in CHOL achieved a perfect value of 1.0.

### Survival prognosis analysis

**Figure 7:**
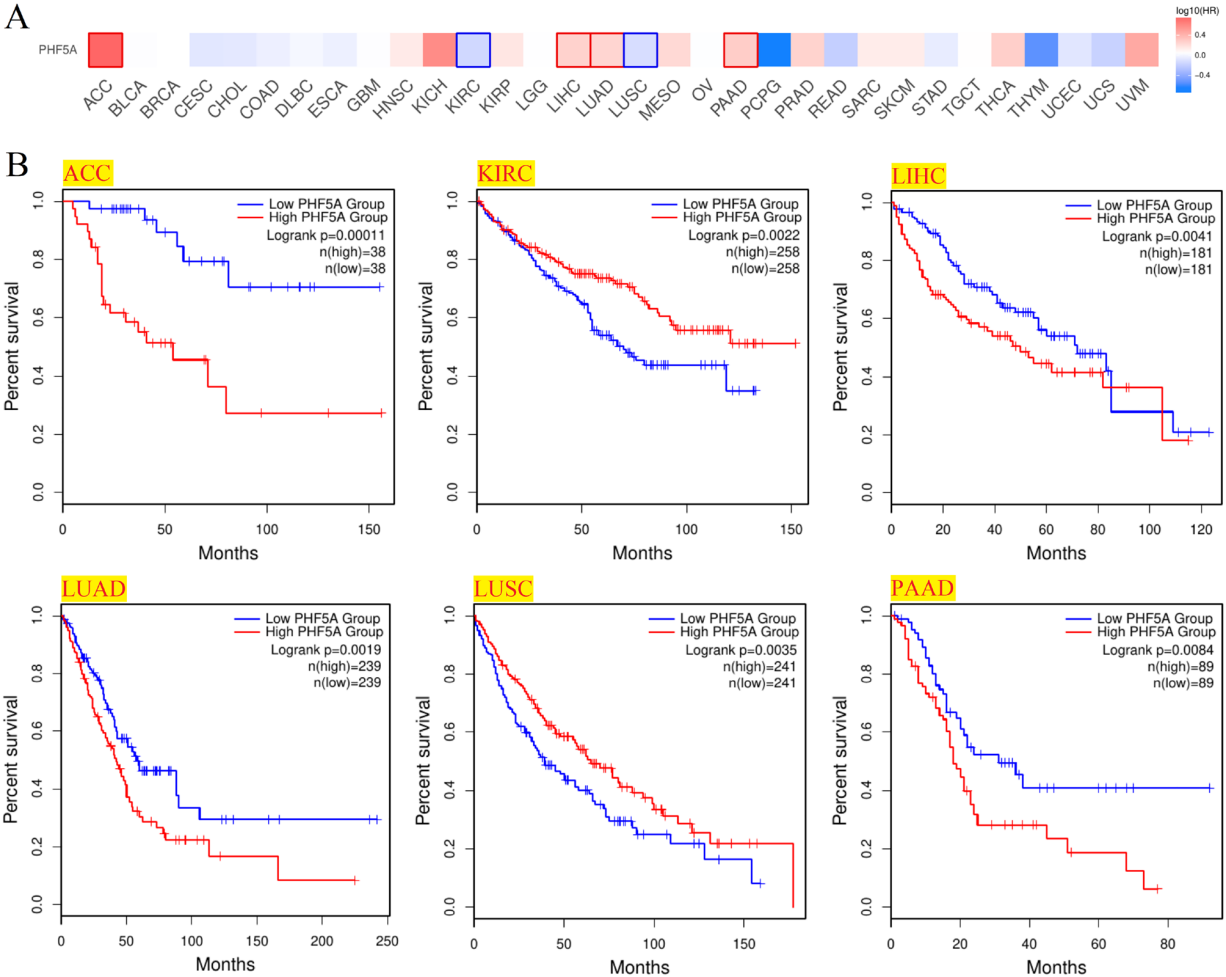
Association of PHF5A expression with patients OS in various cancers using data from TCGA. (**A**) Correlation of patient OS with PHF5A expression in pan-cancer. The solid line framing tumors displayed a remarkable correlation of PHF5A expression with patient OS. (**B**) OS Kaplan–Meier curves in 6 types of tumors with significant differences obtained above.

**Figure 8:**
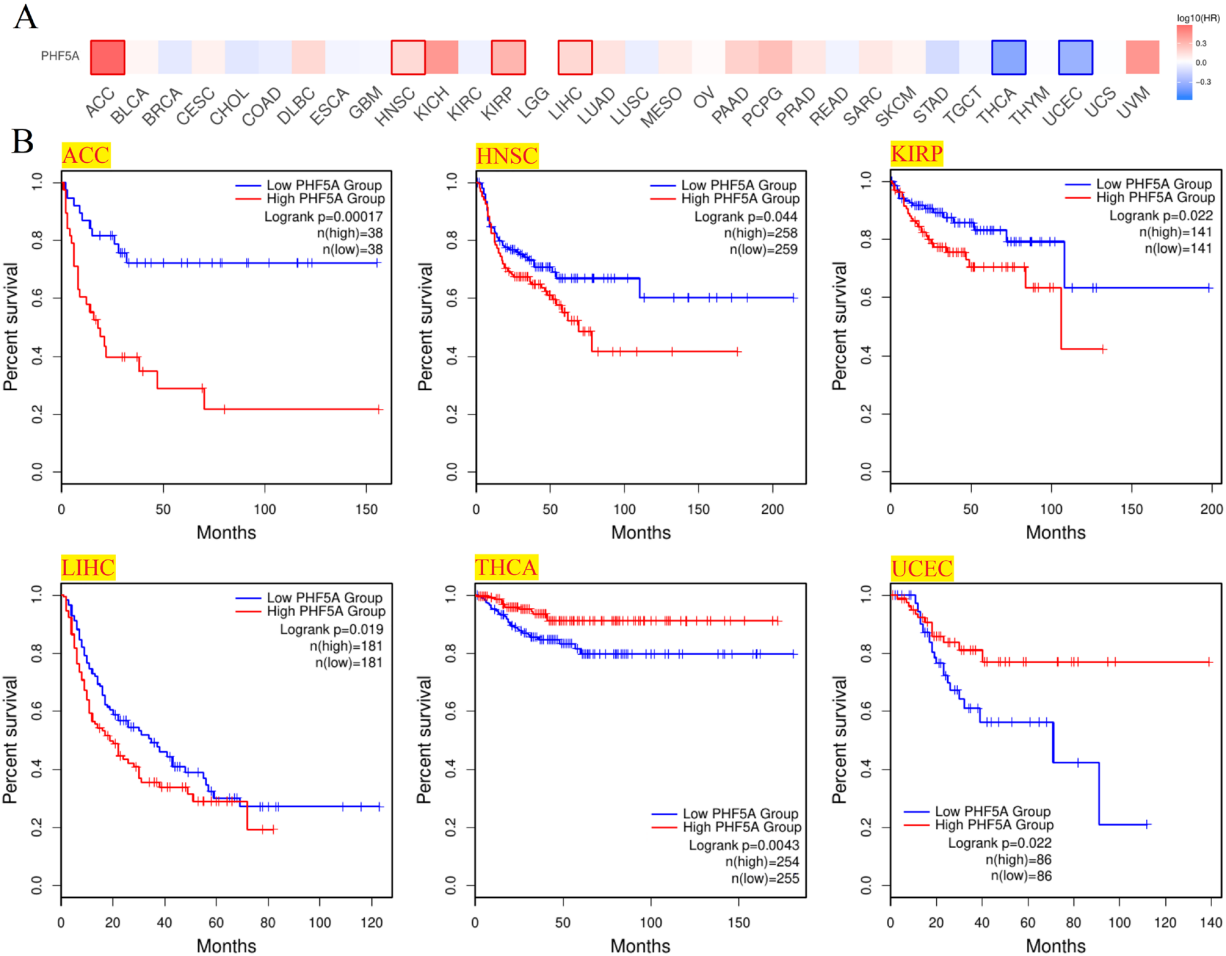
Association of PHF5A expression with patients DFS in various cancers using data from TCGA. (**A**) Correlation of patient DFS with PHF5A expression in pan-cancer. The solid line framing tumors displayed a remarkable correlation of PHF5A expression with patient DFS. (**B**) DFS Kaplan–Meier curves in 6 types of tumors with significant differences obtained above.

Patients with various cancer types were evaluated for their PHF5A expression and prognosis. Patients with low and high expression were equally divided. Notably, Fig. 7A demonstrated a considerably correlation of PHF5A expression with OS prognosis in 6 cancers and Kaplan–Meier curves in these 6 cancers were shown in Fig. 7B. As we could see, in patients with high expression levels of PHF5A, a poorer prognosis for OS was observed in cases of ACC, LIHC, LUAD, as well as PAAD, while in patients with low PHF5A expression, a poorer prognosis for OS was observed in cases of KIRC and LUSC. Additionally, DFS prognosis analysis results (Fig. 8) indicated a significant association between highly expressed PHF5A and a poorer prognosis for ACC, HNSC, KIRP, as well as LIHC, and a better prognosis for THCA and UCEC.

**Figure 9:**
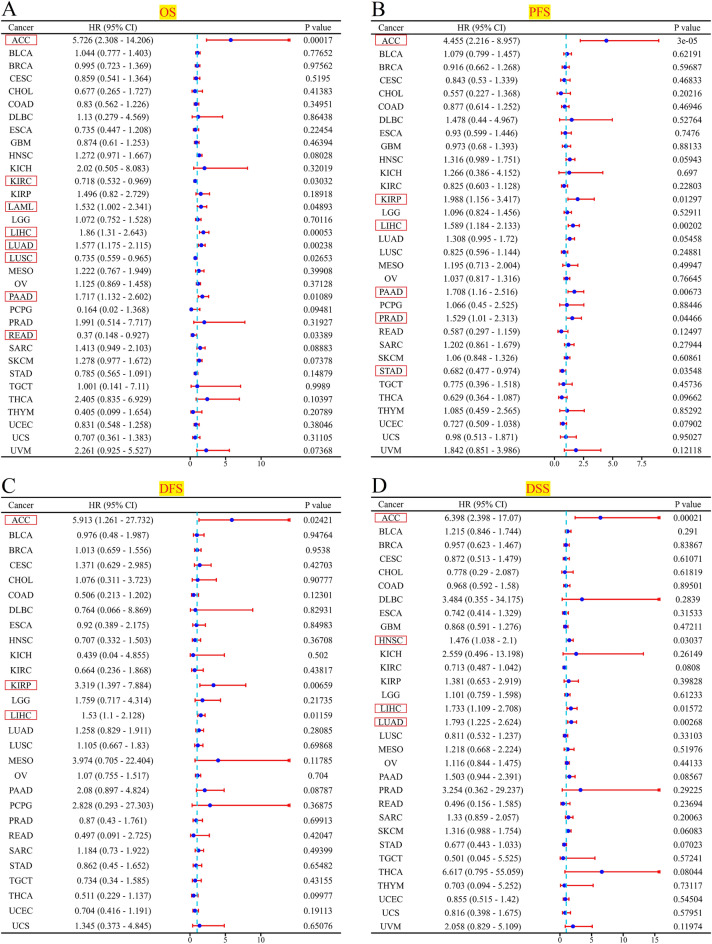
Univariate cox regression analysis of PHF5A. The forest maps demonstrated the univariate cox regression results of PHF5A for OS (**A**), PFS (**B**), DFS (**C**) and DSS (**D**) in multiple tumors. The confidence interval, hazard ratio (HR) and p-value were displayed. The red box displayed results that exhibit significant disparities.

Moreover, we conducted the univariate cox regression analyses utilizing R software and presented the outcomes through the forest map (Fig. 9). Figure 9A revealed that based on the OS findings, PHF5A exhibited a risk effect in ACC, LAML, LIHC, LUAD, as well as PAAD. Conversely, in KIRC, LUSC, and READ, it exhibited a protective effect. The PFS findings indicated that PHF5A exhibited a risk effect in ACC, KIRP, LIHC, PAAD, and PRAD, while it exhibited a protective effect in STAD (Fig. 9B). The DFS findings indicated that PHF5A exhibited a risk effect in patients with ACC, KIRP and LIHC (Fig. 9C). Finally, the DSS findings indicated that PHF5A exhibited a risk effect in ACC, HNSC, LIHC and LUAD (Fig. 9D). The findings showed a significant correlation between patient prognosis and PHF5A expression across various kinds of tumors.

### PHF5A expression level correlates with DNA methylation

**Figure 10:**
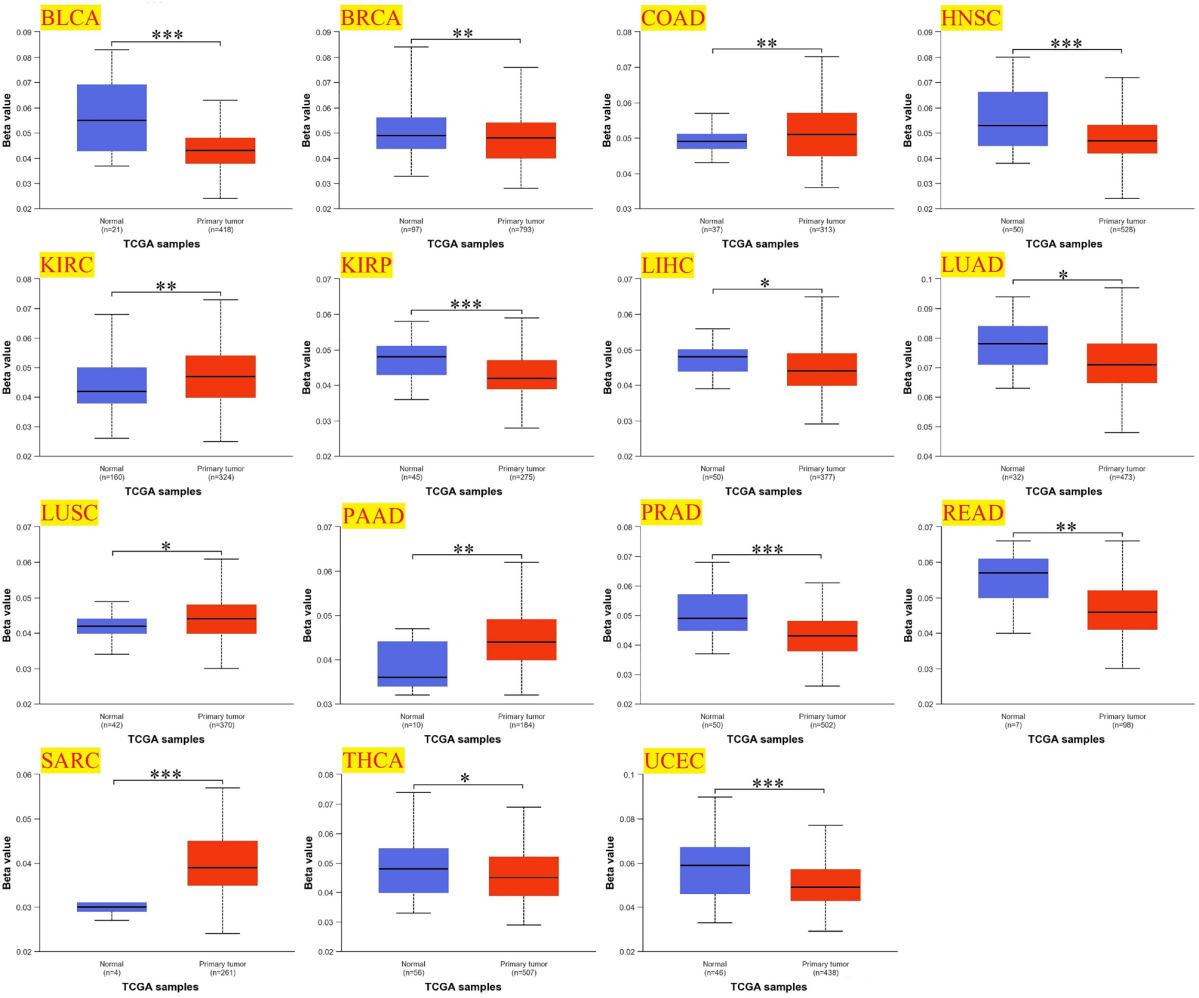
PHF5A promoter methylation level in both primary cancer tissues and normal tissues in various cancers. All significant results from TCGA tumors were presented. Beta value which ranges from 0 to 1 was for characterizing the levels of DNA methylation. *p < 0.05; **p < 0.01; ***p < 0.001.

The occurrence and development of cancer are directly impacted by DNA methylation^31,32^. Figure 10 indicated that PHF5A promoter methylation levels showed significant differences between 15 tumor types compared to normal tissues. In BRCA, BLCA, HNSC, KIRP, LUAD, LIHC, PRAD, READ, THCA as well as UCEC, there was a significant decrease in PHF5A methylation level when compared to normal tissues. However, in COAD, KIRC, LUSC, PAAD, and SARC, the methylation levels of PHF5A were greatly increased.

**Figure 11:**
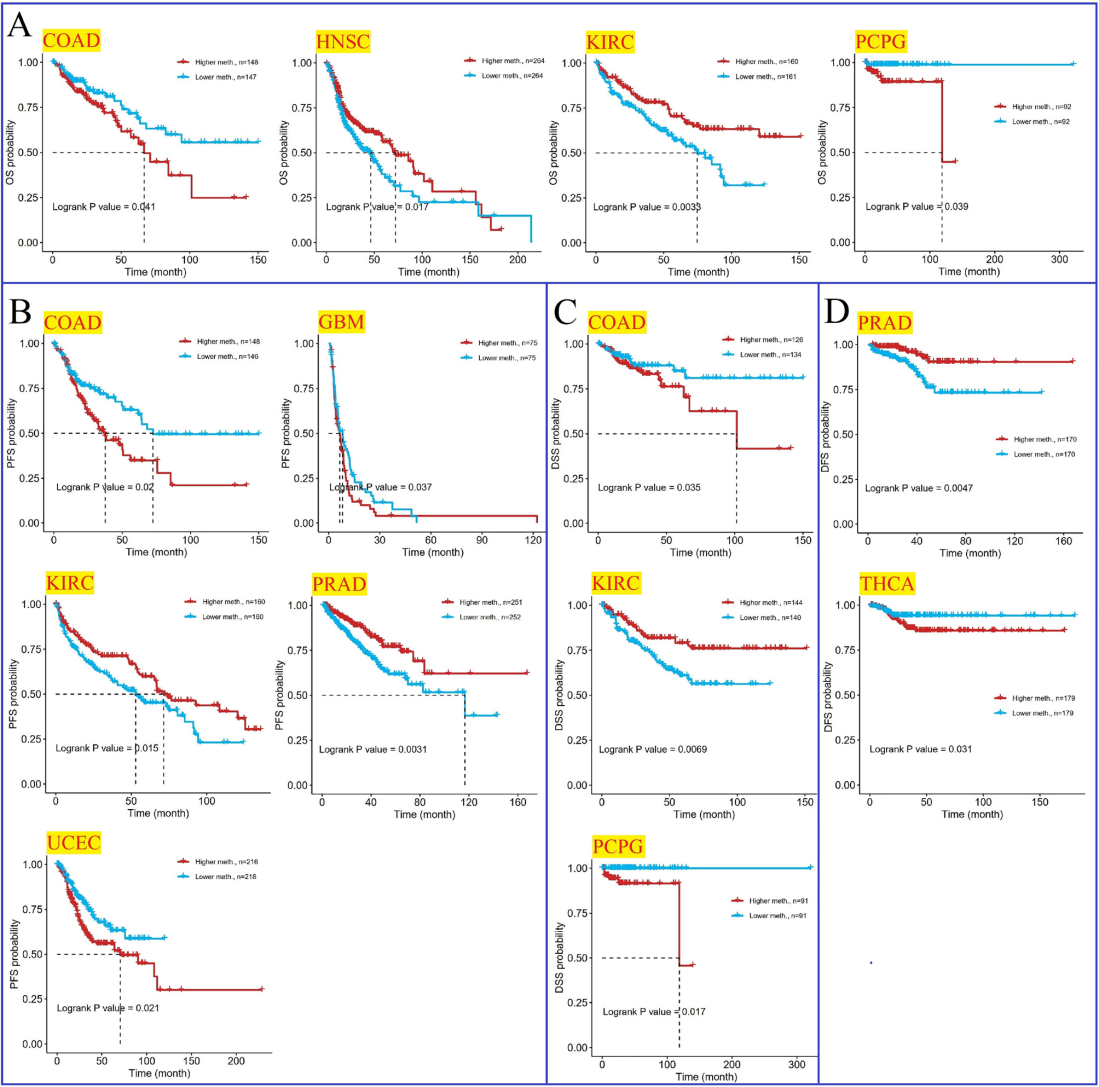
Relationship of PHF5A methylation with patient prognosis in various cancers utilizing data from TCGA. All significant results from TCGA tumors were presented. (**A**) Kaplan–Meier curves demonstrated significant correlation between PHF5A methylation level and OS prognosis in COAD, HNSC, KIRC and PCPG. (**B**) Kaplan–Meier curves demonstrated significant correlation between PHF5A methylation level and PFS prognosis in COAD, GBM, KIRC, PRAD and UCEC. (**C**) Kaplan–Meier curves demonstrated significant correlation between PHF5A methylation level and DSS prognosis in COAD, KIRC and PCPG. (**D**) Kaplan–Meier curves demonstrated significant correlation between PHF5A methylation level and DFS in THCA as well as PRAD.

Moreover, we examined the correlation of patient prognosis with PHF5A promoter methylation. We selected several cancers in which the prognosis was notable linked to PHF5A promoter methylation levels. Figure 11A demonstrated that in terms of OS, PHF5A methylation level played a protective role in HNSC and KIRC, while it exhibited a risk effect in COAD and PCPG. PFS of KIRC and PRAD were positively linked to PHF5A methylation level, but PFS of COAD, GBM and UCEC were negatively linked to PHF5A methylation level (Fig. 11B). Regarding DSS, high PHF5A methylation level was linked to poorer prognosis for COAD and PCPG, while low PHF5A methylation level was linked to poorer prognosis for KIRC (Fig. 11C). Furthermore, in terms of DFS, high PHF5A methylation level was linked to better prognosis for PRAD, while increased PHF5A methylation was associated with unfavorable prognosis in THCA (Fig. 11D).

### Enrichment analysis

**Figure 12:**
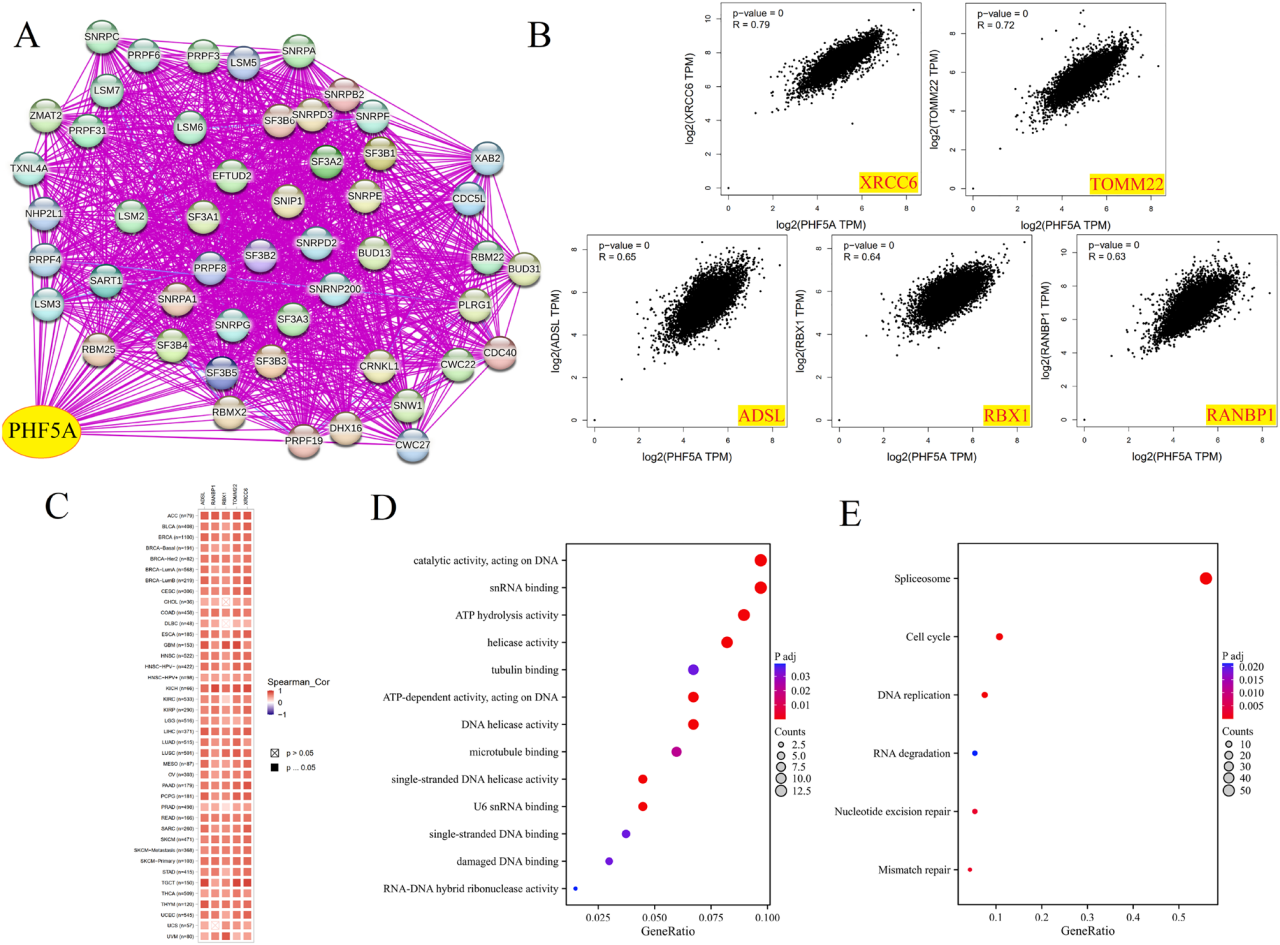
Enrichment analysis of PHF5A-related partners. (**A**) PPI network of proteins that bind to PHF5A though experimental verification. (**B**) Expression relationship between PHF5A and XRCC6, TOMM22, ADSL, RBX1, RANBP1 in TCGA cancers. (**C**) Expression correlation between PHF5A and XRCC6, TOMM22, ADSL, RBX1, RANBP1 in each tumor. (**D**) GO analysis using PHF5A-binding proteins and correlated genes. (**E**) KEGG enrichment analysis using PHF5A-binding proteins and correlated genes.

**Figure 13:**
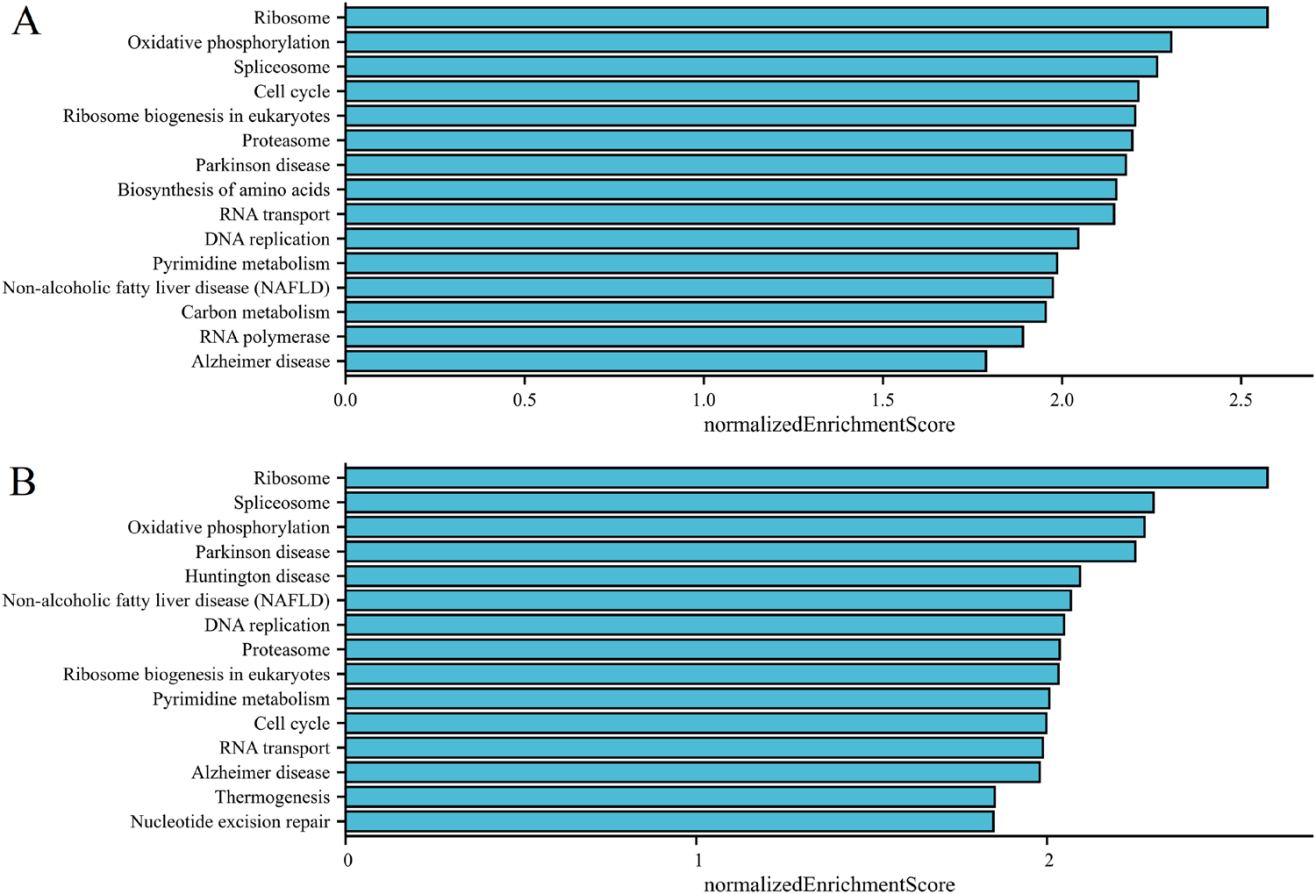
TOP15 GSEA enrichment terms result of PHF5A in the TCGA BRCA (**A**) and HNSC (**B**) cohort using LinkedOmics platform. The KEGG pathways were selected for the GSEA analysis. Normalized enrichment score was represented on horizontal axis while corresponding pathway was represented on vertical axis.

Total of 50 proteins that bind to PHF5A though experimental verification and the top 100 PHF5A expression-correlated genes were acquired to comprehend the involvement of PHF5A in initiation and progression of cancers. Figure 12A showed the interaction network of these proteins (PPI). Figure 12B illustrates the connection between PHF5A and 5 genes (XRCC6, TOMM22, ADSL, RBX1, RANBP1, p < 0.001) that displayed the strongest positive correlation with PHF5A in TCGA cancers. Figure 12C demonstrated that in most cancers, these five genes were positively associated with PHF5A. Then, these two datasets were merged for various analyses pertaining to pathway enrichment. GO enrichment findings demonstrated that the majority of genes were strongly related to molecular function (MF) items of snRNA binding, ATP hydrolysis activity, single-stranded DNA binding, RNA–DNA hybrid ribonuclease activity, single-stranded DNA helicase activity, catalytic activity, acting on DNA, U6 snRNA binding, helicase activity, ATP-dependent activity, acting on DNA, tubulin binding, DNA helicase activity, microtubule binding and damaged DNA binding (Fig. 12D). The KEGG analysis further suggested that the effect of PHF5A on tumors might potentially involve the pathways of spliceosome, nucleotide excision repair, cell cycle, RNA degradation, mismatch repair and DNA replication (Fig. 12E). We further conducted separate evaluations of PHF5A-related signaling pathways in 32 cancers from TCGA using GSEA. The TOP15 GSEA-KEGG enrichment terms in BRCA and HNSC were shown as examples (Fig. 13). The findings revealed a notable relationship between PHF5A and genetic information processing, metabolism and cellular processes, such as Ribosome, spliceosome, ribosome biogenesis in eukaryotes, proteasome, RNA transport, DNA replication, oxidative phosphorylation, pyrimidine metabolism and cell cycle. Thus, we speculated that PHF5A might promote tumor growth by facilitating RNA transcription and degradation, promoting DNA replication and repair and driving the cellular processes.

### Immunological analysis

**Figure 14:**
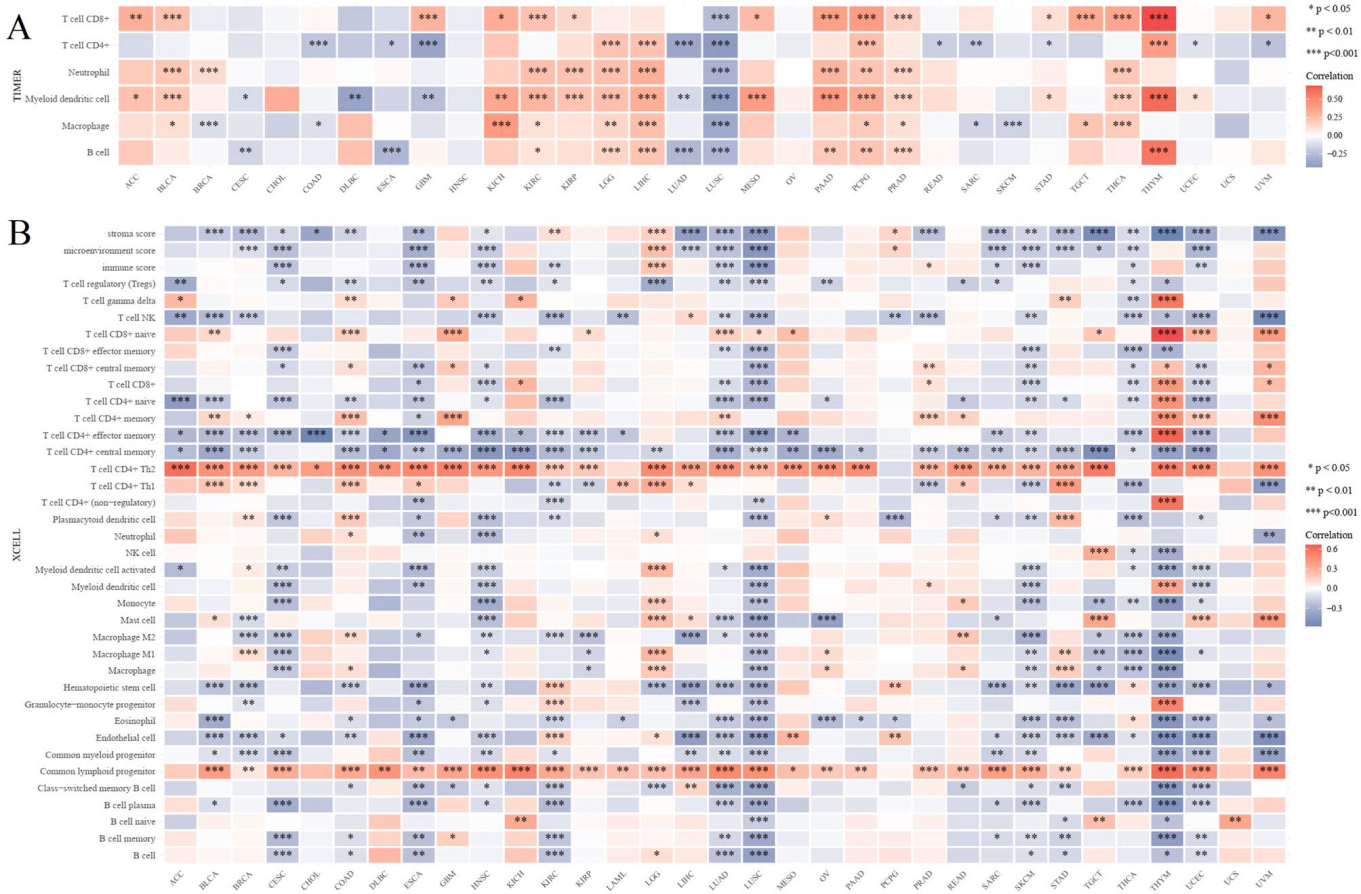
The relationship of immune cell infiltration levels VS PHF5A expression in various cancers. (**A**) PHF5A expression showed a significant correlation against levels of different infiltrating immune cells according to TIMER algorithm. (**B**) PHF5A expression was strongly related to the levels of various infiltrating immune cell subtypes, as determined by xCell algorithm.

The initiation and advancement of cancer is closely associated with tumor-infiltrating immune cells, which have a prominent impact on the tumor microenvironment (TME)^33^. To investigate the involvement of PHF5A in immune response, an extensive analysis was conducted on immune cell infiltration data across different cancer types. Plenty of infiltrating immune cells, including B cells, macrophages, myeloid dendritic cells (DCs), neutrophils, T cells CD4+ and T cells CD8+, were found to have a strong correlation with PHF5A expression in 11, 14, 20, 11, 14, 16 types of tumors respectively (Fig. 14A). The following cancers, namely KIRC, LGG, LIHC, PCPG and PRAD, showed substantial positive correlations with infiltrating immune cells. However, LUSC showed a significant negative correlation with infiltrating immune cells. Furthermore, the correlation of PHF5A expression with various infiltrating immune cell subtypes was investigated using the xCell algorithm. The figure reflected that PHF5A expression was notable linked to infiltrating immune cell levels for majority types of cancer (Fig. 14B). In particular, data showed that PHF5A was significantly and negatively related to majority subtypes in CESC, ESCA, HNSC, LUSC, LUAD, SKCM, THCA, THYM and UCEC. In almost all cancers detected, T cell CD4+ Th2 and common lymphoid progenitor were positively correlated with PHF5A expression, while T cell CD4+ central memory was negatively correlated with it.

**Figure 15:**
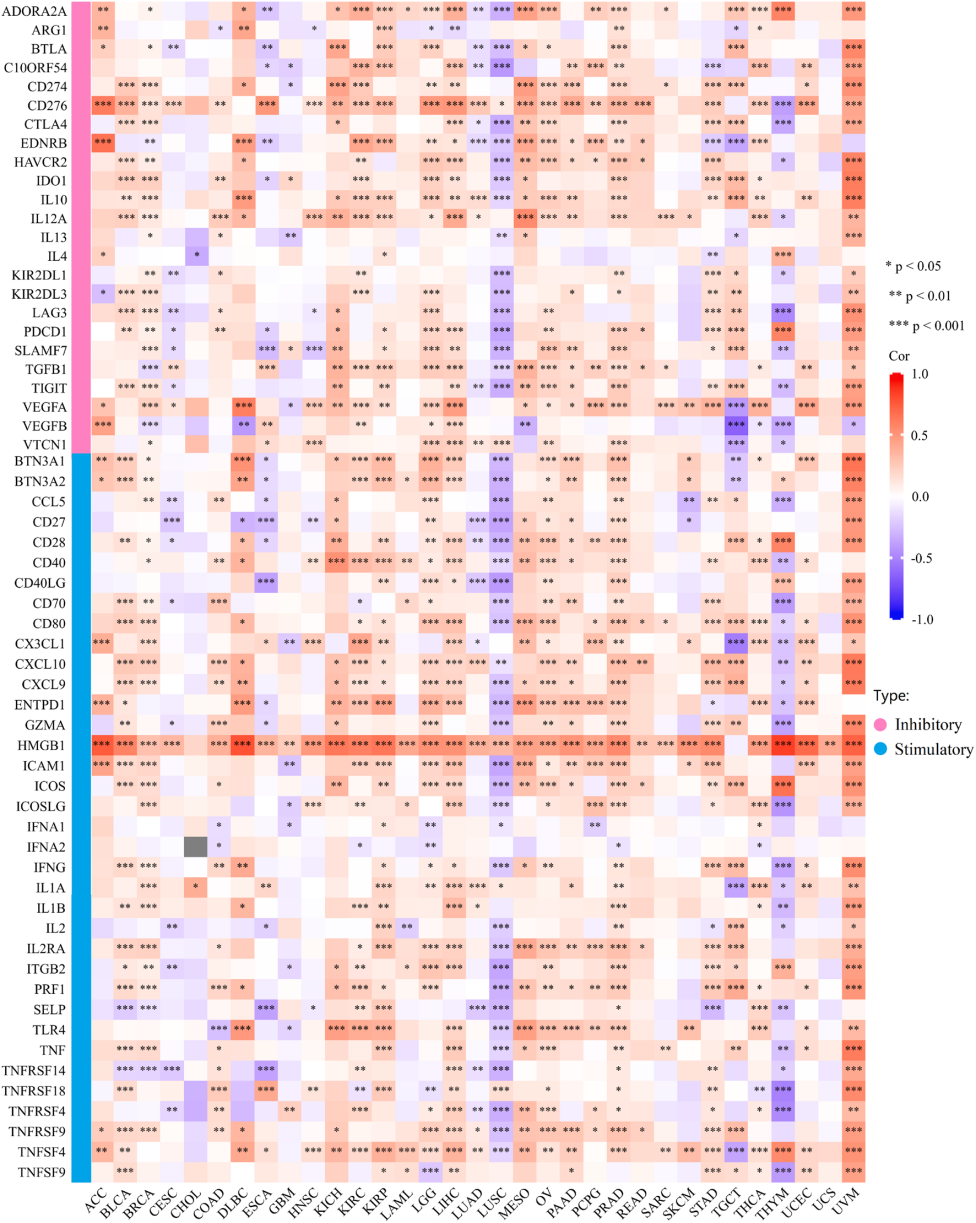
Correlation of immune checkpoint genes with PHF5A expression across various cancers. The immune checkpoint genes can be classified into two types: inhibitory and stimulatory. A close association was found between PHF5A expression and all cancers investigated, except for CHOL and UCS.

**Figure 16:**
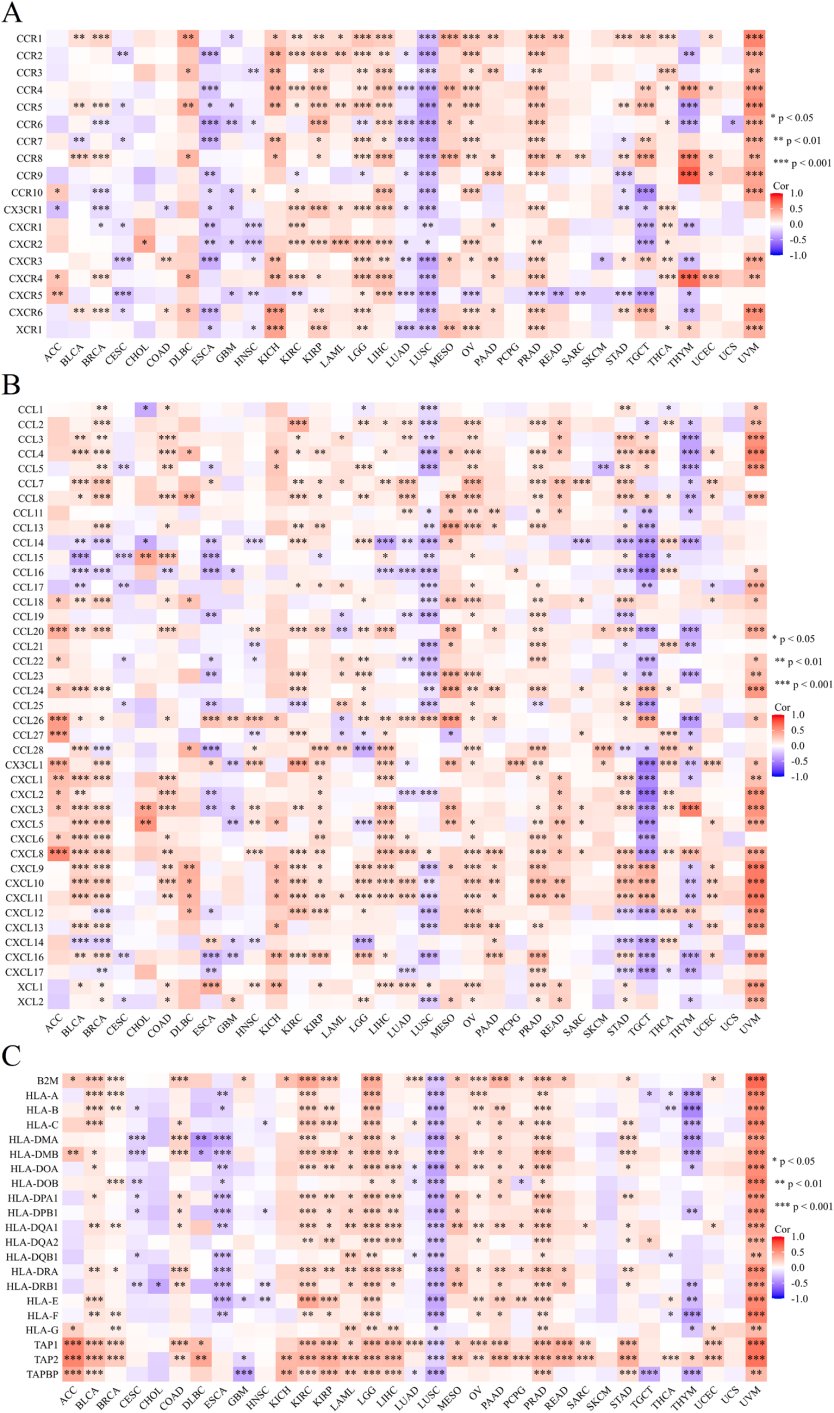
Correlation between PHF5A expression and immunoregulation-related genes: chemokine receptor genes (**A**), chemokine genes (**B**) and MHC genes (**C**). Most of the immunomodulatory genes we investigated exhibited a noteworthy positive correlation with PHF5A.

Owing to expression of immune checkpoint genes is strongly correlated with immune cell infiltration and the effectiveness of immunological therapy^34^, we investigated the correlation between PHF5A expression and 60 genes known as immune checkpoints in various forms of tumor. Notably, a close correlation was found between PHF5A expression and all types of cancer, except for CHOL and UCS (Fig. 15). It was evident that there was a notable positive correlation between PHF5A expression and most immune checkpoint genes in the majority of tumors, except for CESC, ESCA, LUSC, and THYM. Among the 60 immune checkpoint genes, PHF5A expression was found to be significantly positively correlated with 53 genes in PRAD and 50 genes in UVM. However, in LUSC, PHF5A expression showed a significantly negative correlation with 45 genes. Furthermore, we determined the association of PHF5A expression with chemokine receptors, chemokines and MHC genes in pan-cancer. Figure 16 showed that almost all the immunomodulatory genes we investigated were related to the expression of PHF5A, and most of them exhibited a noteworthy positive correlation with PHF5A in cancers. These indications suggested that PHF5A could be a perfect candidate for immunotherapy.

**Figure 17:**
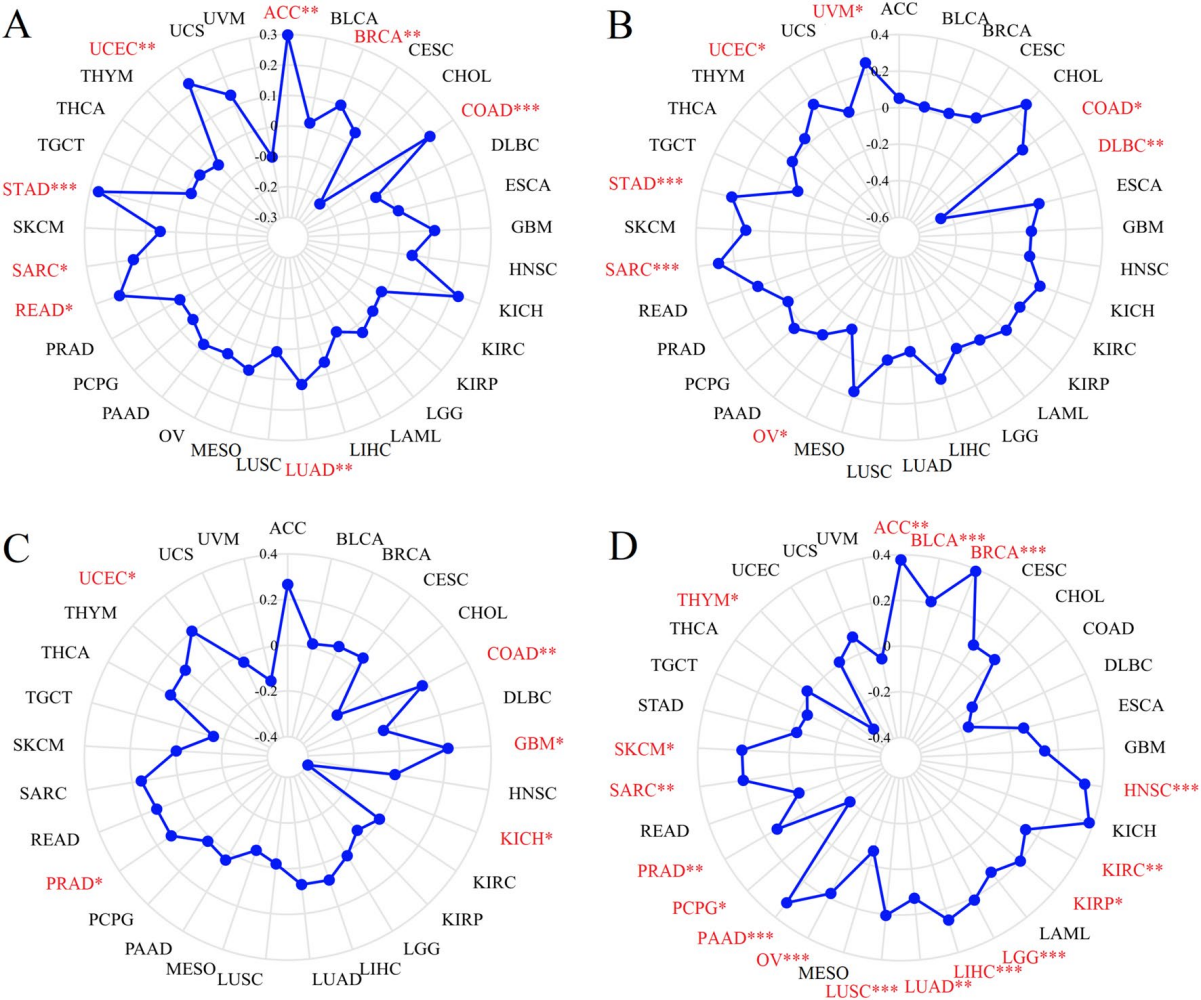
The relationship of PHF5A expression with TMB (**A**), MSI (**B**), NEO (**C**) and HRD (**D**) in multiple cancers. The red font indicated results that showed significant differences. *p < 0.05; **p < 0.01; ***p < 0.001.

Antitumor immunity serves as a reliable predictor of tumor immunotherapy efficacy, closely connected to TMB, MSI, NEO, and HRD^35–38^. Thus, the correlations between PHF5A expression and TMB, MSI, NEO and HRD were investigated to ascertain whether PHF5A can predict immunotherapeutic responses. Results were shown in Fig. 17 and the points represented the correlation coefficient. In BRCA, LUAD, SARC, COAD, READ, UCEC, STAD and ACC, a notable positive association was observed between PHF5A expression and TMB (Fig. 17A). In UCEC, SARC, STAD, UVM and COAD, a significant positive correlation of PHF5A expression with MSI was observed (Fig. 17B). Conversely, in OV and DLBC, a notable negative correlation of PHF5A expression with MSI was observed. Furthermore, Fig. 17C demonstrated that PHF5A expression was notably negatively related to NEO in KICH. Conversely, in PRAD, UCEC, COAD and GBM, a notable positive correlation of NEO with PHF5A expression was observed. Furthermore, PHF5A expression was notably positively related to HRD in LUAD, PRAD, KIRC, OV, KIRP, LUSC, SKCM, BLCA, SARC, LGG, LIHC, PAAD, HNSC, ACC, and BRCA (Fig. 17D). Additionally, a significant negative association with HRD was observed in PCPG and THYM. The above results verified that the regulation of PHF5A on the composition and immune mechanism within the TME might impact anti-tumor immunity. Accordingly, it can be inferred that PHF5A has the capacity to act as an immunotherapy target or biomarker for different types of cancer, and can also function as an indicator for the effectiveness of tumor immunotherapy.

### Drug sensitivity analysis

**Figure 18:**
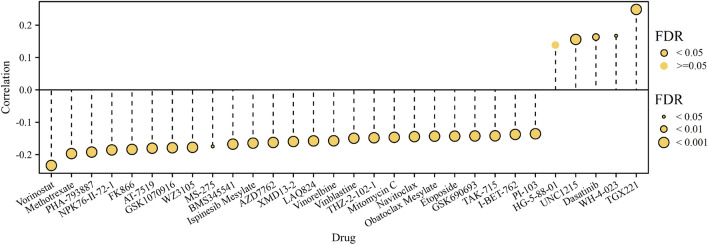
Correlation of PHF5A expression with GDSC drugs sensitivity in pan-cancer. Top 30 GDSC drugs exhibiting the strongest correlation with PHF5A expression were presented. Black outline border manifested FDR ≤ 0.05. Bubble size positively related to the significance of FDR.

GSCA platform was utilized to investigate correlation of PHF5A expression with anti-tumor drugs sensitivity in pan-cancer. This study identified the top 30 GDSC drugs that exhibited strongest correlation with PHF5A expression, as depicted in Fig. 18. It was evident that the sensitivity of 29 out of 30 drugs was notably correlated with PHF5A expression, such as TGX221, vorinostat, methotrexate, PHA-793887 and NPK76-II-72-1. Specifically, the sensitivity of 4 drugs demonstrated a significantly positive correlation with PHF5A expression, while the sensitivity of the remaining 25 drugs exhibited a significantly negative correlation with PHF5A expression.

## Discussion

Cancer is a common health problem faced by humans, and the incidence and mortality of cancers are increasing rapidly in the world^1^, and more researches are needed for the mechanisms of cancer occurrence and progression to get a radical treatment. The pan-cancer analysis helps to identify gene of interest in various types of cancer, utilize database to explore possible molecular mechanisms, and thus provide deep insights for treatment. PHF5A has been proven to be associated with transcription and splicing activities in previous studies, and an increasing amount of evidence indicates that it has a significant influence on the initiation and advancement of various types of cancers. For example, PHF5A plays a significant role in migrating and invading hepatocellular carcinoma cells^13^, and in breast cancer, PHF5A expression is elevated and effects a lot in tumor formation^5^. Knockdown of PHF5A can inhibit malignant transformation of gastric cancer cell lines, and experiments have shown that the AKT/mTOR signaling pathway works well in this process^14^, while in colorectal cancer, the over-expressed PHF5A has been shown to promote the cancer cells growth and transfer^15^. Here, the action of PHF5A in various cancers was systematically evaluated using multiple public databases.

The PHF5A expression is elevated in many types of cancer, such as breast cancer, colorectal cancer, gastric cancer and hepatocellular carcinoma. This elevation acts a significant role in the onsets and progress of these cancers^5,13–15^. In this finding, mRNA expression analysis indicated that PHF5A exhibited a notably elevated levels in 21 types of cancer (Fig. 1). Additionally, protein level expression and the immunohistochemical analysis provided further evidence support (Fig. 3). This finding aligned with previous studies, and these results implied that PHF5A held great promise in clinical diagnosis. Moreover, molecular subtypes showed differential expression of PHF5A in the majority of cancers (Fig. 4). This result might provide insights for future studies on the underlying mechanism of PHF5A in cancer. The link of tumor clinical stages with PHF5A expression were also analyzed thoroughly. Research on lung adenocarcinoma has indicated that PHF5A expression is positively correlated with the T stage as well as lymph node metastasis. Additionally, PHF5A has been found to be necessary for the invasion and migration of lung adenocarcinoma cells^39^. Previous studies reveal that increased expression of PHF5A in colorectal cancer is associated with advanced TNM stage and the metastasis of lymph node^15^. Here, the present results revealed that PHF5A expression was linked to main pathological stages of 3 types of tumors, and was associated with 2 cancers in the M stage and 6 cancers in the N stage (Fig. 2). Among them, PHF5A expression was found to be related to the N stage in LUAD and was also linked to the main pathological stage as well as the N stage in COAD. Those findings were similar to the results observed in lung adenocarcinoma and colorectal cancer^15,39^. Additionally, we noticed that PHF5A expression showed a correlation with the grade of 6 different tumor types. It is important to note that the tumor grade increased correspondingly with the increasing expression of PHF5A.

As is well-known, cancer detection has important clinical significance and related researches has promoted the frontier of cancer diagnosis. The significance of PHF5A for diagnosis has been mentioned in research, which indicates that acetylation of PHF5A-K29 can potentially serve as a diagnostic biomarker for colorectal cancer^11^. Here, PHF5A showcased remarkable performance in diagnosing multiple cancers, as confirmed in Fig. 6. The results showed that PHF5A could be used in the diagnosis of 21 types of cancer, and an important point to note was that AUC in CHOL achieved a perfect value of 1.0. As we can see, PHF5A had diagnostic significance for both COAD and READ, with a diagnostic accuracy of 0.835 and 0.685, respectively. This result echoes previous research findings^11^. Our results once again confirmed that PHF5A held great promise in tumor diagnosis.

Previous results have shown that the role of PHF5A is associated with various tumors. Research has implied that PHF5A is involved in the progression of colorectal carcinoma^11^. Similarly, for breast cancer, PHF5A is characterized as a promoter, and serves as a strong indicator of a decreased likelihood of patient survival after surgery^5^. Moreover, PHF5A is also a novel diagnostic and prognostic marker in LUAD^39^. We have conducted a comprehensive evaluation of patients’ prognosis by utilizing Kaplan–Meier analysis and univariate cox regression analysis (Figs. 7, 8, 9). The findings hinted that high PHF5A expression was predominantly connected with increased risk in different kinds of tumor and PHF5A could be a valuable and potential biomarker with significant implications in prognosis. In addition, we noticed that the higher PHF5A expression in these cancers basically corresponded to the higher stage or grade of cancer (Fig. 2). Results demonstrated that high PHF5A expression posed a risk in all four prognostic types (OS, DFS, DFS and DSS) of ACC and LIHC. Higher clinicopathological stage in ACC was found to be correlated with increased expression of PHF5A, as indicated by clinical staging results. Additionally, the elevated expression of PHF5A in ACC was found to be significantly associated with the presence of distant metastasis and an increased occurrence of lymph node metastasis. In LIHC, as the expression of PHF5A increased, the tumor grade also elevated. A similar pattern could be found in other cancers, such as LUAD, PAAD, and HNSC.

DNA methylation, a common epigenetic modification of DNA, possesses the capability to regulate genetic expression with no alterations in DNA sequencing^31^. In recent decades, scientists have gradually discovered the connections between DNA methylation and cancer. The current research revealed a notable disparity in PHF5A promoter methylation levels between 15 kinds of tumor and the normal tissues. Reduced PHF5A methylation level was observed in 10 types of tumors, including those in which PHF5A was mentioned as a risk factor, such as LIHC, LUAD, KIRP, PRAD and HNSC. On the other hand, the opposite trend was observed in the remaining 5 types of cancer, including those in which PHF5A acted as a protective factor, such as KIRC and LUSC (Fig. 10). The findings suggested that the body may regulate the active expression of PHF5A by controlling its methylation levels, and DNA methylation played a role in the action of PHF5A in cancers. Additionally, the analysis of the correlation between methylation of PHF5A promoter and prognosis has been firstly conducted, and we found that promoter methylation of PHF5A played a guiding role in prognosis in some cancers (Fig. 11).

We conducted various analyses pertaining to pathway enrichment. PHF5A, functioning as a splicing factor, has been shown to significantly impact several cancer types, such as breast cancer^5^, lung cancer^40^, and rectal cancer^11,15^. Moreover, PHF5A plays a role in cell growth and differentiation^41^, DNA damage repair^10^, chromatin remodeling^7,8^ and pluripotency maintenance of embryonic stem cells^9^. The examination indicated that PHF5A may impact tumor development through the involvement of spliceosome, ribosome, proteasome, DNA replication, mismatch repair, cell cycle, RNA degradation, and transport pathways (Figs. 12, 13). The presented result was in line with the findings of earlier literatures. Those findings revealed that the abnormal function of PHF5A was correlated with the malignant behaviors of cancer cells and PHF5A might be a novel anti-tumor target.

So far, there is limited information available regarding the involvement of PHF5A in immune system of tumor patients, and further research on the role of PHF5A in tumor immunity remains an area worth exploring. Our findings showed that PHF5A expression varied significantly among various immune subtypes in majority of tumors (Fig. 5) and suggested that PHF5A might act a role in immune regulation. The onsets and progress of cancer is significantly influenced by immune cells that infiltrate the tumor^42^, and also the TME is regulated by various immune cell types. Having a thorough comprehension regarding the immune infiltration condition of tumor patients is especially crucial in order to choose the appropriate personalized immunotherapy approach^43,44^. Our study revealed that a notable association was observed between PHF5A expression and the levels of different infiltrating immune cells and their subtypes, including B cells, macrophages, DCs, neutrophils, T cells CD8+ and T cells CD4+, in many kinds of tumors (Fig. 14). However, the correlation of immune cells with PHF5A expression might vary in various cancers, indicating that PHF5A might have distinct immunomodulatory functions in different cancers. Moreover, PHF5A expression was related to the examined immunoregulation-related genes of chemokine receptor, chemokine and MHC, which further demonstrated its potential immune function in different kinds of tumor (Fig. 16). The findings suggested a close relationship of PHF5A expression with immune infiltration of tumor cells, indicating its role in regulating tumor immunity across different types of tumors. Besides, the reliable prediction of immunotherapy effectiveness in different cancers is often based on immune-related genes expression^45–48^. The utilization of immune checkpoint gene expression is employed to identify patients with tumors who are expected to derive significant benefits from immune checkpoint blockade therapy^42^. In multiple cancers, there was a notable connection found between PHF5A and immune checkpoint genes, where PHF5A expression demonstrated a positive association with a majority of immune checkpoint genes (Fig. 15). The results provided a theoretical foundation for future molecular targeted immunotherapy. Recent research findings have demonstrated that immunotherapy is more successful in treating tumors with high MSI and TMB^49,50^. NEO have been identified as potential targets for tumor immunotherapy using T cells^51,52^ and the status of HRD acts as a vital indicator for different tumor treatment choices and prognosis^53,54^. In various tumors, our findings indicated a strong correlation of PHF5A expression with TMB, MSI, NEO and HRD (Fig. 17). PHF5A expression level might potentially affect patients’ response to immune checkpoint therapy. Enhancing our comprehension of the immunotherapy’s mechanism of action in tumor treatment will offer a fresh point of reference for predicting its prognosis. Consequently, it can be inferred that PHF5A has the capacity to act as an immunotherapy target or biomarker for different types of cancer, and can also function as an indicator for the effectiveness of tumor immunotherapy.

Drug therapy is a widely utilized approach for cancer treatment, and investigating the correlation between drug sensitivity and biomarkers matters a lot. Our findings revealed a notable correlation of PHF5A expression with sensitivity of various anti-tumor drugs (Fig. 18). This suggested that PHF5A might be involved in chemotherapy and its potential association with drug resistance.

To summarize, the present investigation confirmed that the PHF5A expression was elevated in the majority of tumors, emphasizing a significance of PHF5A expression for the clinical assessment and prognosis of different types of cancers. Our research findings clearly demonstrated the importance of PHF5A in tumor immunity, highlighting the potential of PHF5A as a key candidate for anti-tumor immunotherapy. The discovery could potentially enhance our overall comprehension of the function of PHF5A in the initiation and advancement of tumors. For the foreseeable future, they could serve as a valuable reference for advancing individualized immunotherapy. In short, PHF5A is a potential diagnostic, prognostic, and immunological biomarker in pan-cancer.

## Data Availability

All data comes from public databases. Links to these databases have been provided in the article.

## References

[1] Sung H, Ferlay J, Siegel RL (2021). Global Cancer Statistics 2020: GLOBOCAN estimates of incidence and mortality worldwide for 36 cancers in 185 countries. CA Cancer J. Clin..

[2] Siegel RL, Miller KD, Jemal A (2019). Cancer statistics, 2019. CA Cancer J. Clin..

[3] Hanahan D (2022). Hallmarks of cancer: New dimensions. Cancer Discov..

[4] Chatterjee A, Rodger EJ, Eccles MR (2018). Epigenetic drivers of tumourigenesis and cancer metastasis. Semin. Cancer Biol..

[5] Zheng YZ, Xue MZ, Shen HJ (2018). PHF5A epigenetically inhibits apoptosis to promote breast cancer progression. Cancer Res..

[6] Falck E, Klinga-Levan K (2013). Expression patterns of Phf5a/PHF5A and Gja1/GJA1 in rat and human endometrial cancer. Cancer Cell Int..

[7] Trappe R, Ahmed M, Gläser B (2002). Identification and characterization of a novel murine multigene family containing a PHD-finger-like motif. Biochem. Biophys. Res. Commun..

[8] Trappe R, Schulze E, Rzymski T, Fröde S, Engel W (2002). The *Caenorhabditis elegans* ortholog of human PHF5a shows a muscle-specific expression domain and is essential for *C. elegans* morphogenetic development. Biochem. Biophys. Res. Commun..

[9] Strikoudis A, Lazaris C, Trimarchi T (2016). Regulation of transcriptional elongation in pluripotency and cell differentiation by the PHD-finger protein Phf5a. Nat. Cell Biol..

[10] Begum NA, Haque F, Stanlie A (2021). Phf5a regulates DNA repair in class switch recombination via p400 and histone H2A variant deposition. EMBO J..

[11] Wang Z, Yang X, Liu C (2019). Acetylation of PHF5A modulates stress responses and colorectal carcinogenesis through alternative splicing-mediated upregulation of KDM3A. Mol. Cell.

[12] Hubert CG, Bradley RK, Ding Y (2013). Genome-wide RNAi screens in human brain tumor isolates reveal a novel viability requirement for PHF5A. Genes Dev..

[13] Yang Q, Zhang J, Xu S (2019). Knockdown of PHF5A inhibits migration and invasion of HCC cells via downregulating NF-κB signaling. Biomed. Res. Int..

[14] Zhang Z, Peng L, Yang W, Li B, Hua Y, Luo S (2023). PHF5A facilitates the development and progression of gastric cancer through SKP2-mediated stabilization of FOS. J. Transl. Med..

[15] Chang Y, Zhao Y, Wang L (2021). PHF5A promotes colorectal cancer progression by alternative splicing of TEAD2. Mol. Ther. Nucleic Acids.

[16] Li T, Fu J, Zeng Z (2020). TIMER2.0 for analysis of tumor-infiltrating immune cells. Nucleic Acids Res..

[17] Tang Z, Kang B, Li C, Chen T, Zhang Z (2019). GEPIA2: An enhanced web server for large-scale expression profiling and interactive analysis. Nucleic Acids Res..

[18] Yu Y, Sun Y, Li Z, Li J, Tian D (2023). Systematic analysis identifies XRCC4 as a potential immunological and prognostic biomarker associated with pan-cancer. BMC Bioinform..

[19] Chandrashekar DS, Karthikeyan SK, Korla PK (2022). UALCAN: An update to the integrated cancer data analysis platform. Neoplasia.

[20] Chen F, Chandrashekar DS, Varambally S, Creighton CJ (2019). Pan-cancer molecular subtypes revealed by mass-spectrometry-based proteomic characterization of more than 500 human cancers. Nat. Commun..

[21] Uhlén M, Fagerberg L, Hallström BM (2015). Proteomics. Tissue-based map of the human proteome. Science.

[22] Ru B, Wong CN, Tong Y (2019). TISIDB: An integrated repository portal for tumor-immune system interactions. Bioinformatics.

[23] Smoot BJ, Wong JF, Dodd MJ (2011). Comparison of diagnostic accuracy of clinical measures of breast cancer-related lymphedema: Area under the curve. Arch. Phys. Med. Rehabil..

[24] Robin X, Turck N, Hainard A (2011). pROC: An open-source package for R and S+ to analyze and compare ROC curves. BMC Bioinform..

[25] Xiong Y, Yuan L, Xiong J (2020). An outcome model for human bladder cancer: A comprehensive study based on weighted gene co-expression network analysis. J. Cell Mol. Med..

[26] Liu CJ, Hu FF, Xia MX, Han L, Zhang Q, Guo AY (2018). GSCALite: A web server for gene set cancer analysis. Bioinformatics.

[27] Szklarczyk D, Gable AL, Nastou KC (2021). The STRING database in 2021: Customizable protein–protein networks, and functional characterization of user-uploaded gene/measurement sets [published correction appears in Nucleic Acids Res. 2021 Oct 11;49(18):10800]. Nucleic Acids Res..

[28] Wu T, Hu E, Xu S (2021). ClusterProfiler 4.0: A universal enrichment tool for interpreting omics data. Innovation (Camb.).

[29] Vasaikar SV, Straub P, Wang J, Zhang B (2018). LinkedOmics: Analyzing multi-omics data within and across 32 cancer types. Nucleic Acids Res..

[30] Sturm G, Finotello F, List M (2020). Immunedeconv: An R package for unified access to computational methods for estimating immune cell fractions from bulk RNA-sequencing data. Methods Mol. Biol..

[31] Mehdi A, Rabbani SA (2021). Role of methylation in pro- and anti-cancer immunity. Cancers (Basel).

[32] Kulis M, Esteller M (2010). DNA methylation and cancer. Adv. Genet..

[33] Fridman WH, Galon J, Dieu-Nosjean MC (2011). Immune infiltration in human cancer: Prognostic significance and disease control. Curr. Top. Microbiol. Immunol..

[34] Topalian SL, Drake CG, Pardoll DM (2015). Immune checkpoint blockade: A common denominator approach to cancer therapy. Cancer Cell.

[35] Picard E, Verschoor CP, Ma GW, Pawelec G (2020). Relationships between immune landscapes, genetic subtypes and responses to immunotherapy in colorectal cancer. Front. Immunol..

[36] Choucair K, Morand S, Stanbery L, Edelman G, Dworkin L, Nemunaitis J (2020). TMB: A promising immune-response biomarker, and potential spearhead in advancing targeted therapy trials. Cancer Gene Ther..

[37] van Velzen MJM, Derks S, van Grieken NCT, Haj Mohammad N, van Laarhoven HWM (2020). MSI as a predictive factor for treatment outcome of gastroesophageal adenocarcinoma. Cancer Treat. Rev..

[38] Peng M, Mo Y, Wang Y (2019). Neoantigen vaccine: An emerging tumor immunotherapy. Mol. Cancer.

[39] Yang Y, Zhu J, Zhang T (2018). PHD-finger domain protein 5A functions as a novel oncoprotein in lung adenocarcinoma. J. Exp. Clin. Cancer Res..

[40] Mao S, Li Y, Lu Z (2019). PHD finger protein 5A promoted lung adenocarcinoma progression via alternative splicing. Cancer Med..

[41] Oltra E, Verde F, Werner R, D’Urso G (2004). A novel RING-finger-like protein Ini1 is essential for cell cycle progression in fission yeast. J. Cell Sci..

[42] Jia Q, Chiu L, Wu S (2020). Tracking neoantigens by personalized circulating tumor DNA sequencing during checkpoint blockade immunotherapy in non-small cell lung cancer. Adv. Sci. (Weinh.).

[43] Zhang Y, Zhang Z (2020). The history and advances in cancer immunotherapy: Understanding the characteristics of tumor-infiltrating immune cells and their therapeutic implications. Cell Mol. Immunol..

[44] Murciano-Goroff YR, Warner AB, Wolchok JD (2020). The future of cancer immunotherapy: Microenvironment-targeting combinations. Cell Res..

[45] Xiao Y, Yu D (2021). Tumor microenvironment as a therapeutic target in cancer. Pharmacol. Ther..

[46] Ren D, Hua Y, Yu B (2020). Predictive biomarkers and mechanisms underlying resistance to PD1/PD-L1 blockade cancer immunotherapy [published correction appears in Mol Cancer. 2020 Feb 14;19(1):31]. Mol. Cancer.

[47] He X, Xu C (2020). Immune checkpoint signaling and cancer immunotherapy. Cell Res..

[48] Hu J, Yu A, Othmane B (2021). Siglec15 shapes a non-inflamed tumor microenvironment and predicts the molecular subtype in bladder cancer. Theranostics.

[49] Goodman AM, Sokol ES, Frampton GM, Lippman SM, Kurzrock R (2019). Microsatellite-stable tumors with high mutational burden benefit from immunotherapy. Cancer Immunol. Res..

[50] Jardim DL, Goodman A, de Melo GD, Kurzrock R (2021). The challenges of tumor mutational burden as an immunotherapy biomarker. Cancer Cell.

[51] Schumacher TN, Schreiber RD (2015). Neoantigens in cancer immunotherapy. Science.

[52] Li L, Goedegebuure SP, Gillanders WE (2017). Preclinical and clinical development of neoantigen vaccines. Ann. Oncol..

[53] Miller RE, Leary A, Scott CL (2020). ESMO recommendations on predictive biomarker testing for homologous recombination deficiency and PARP inhibitor benefit in ovarian cancer. Ann. Oncol..

[54] Zhou Z, Ding Z, Yuan J (2022). Homologous recombination deficiency (HRD) can predict the therapeutic outcomes of immuno-neoadjuvant therapy in NSCLC patients. J. Hematol. Oncol..

